# ZIKV infection effects changes in gene splicing, isoform composition and lncRNA expression in human neural progenitor cells

**DOI:** 10.1186/s12985-017-0882-6

**Published:** 2017-11-07

**Authors:** Benxia Hu, Yongxia Huo, Liping Yang, Guijun Chen, Minhua Luo, Jinlong Yang, Jumin Zhou

**Affiliations:** 1 Key Laboratory of Animal Models and Human Disease Mechanisms of the Chinese Academy of Sciences/Key Laboratory of Bioactive Peptides of Yunnan Province, Kunming Institute of Zoology, Kunming, 650223 China; 2 Kunming College of Life Science, University of Chinese Academy of Sciences, Kunming, 650204 China; 3 State Key Laboratory of Virology, CAS Center for Excellence in Brain Science and Intelligence Technology (CEBSIT), Wuhan Institute of Virology, Wuhan, 430071 China; 4 BGI-Yunnan, BGI-Shenzhen, Kunming, 650000 China; 5 College of Life Sciences, Yunnan University, Kunming, 650091 China

**Keywords:** lncRNA, Gene isoform, Alternative splicing, hNPC, ZIKV

## Abstract

**Background:**

The Zika virus (ZIKV) is a mosquito-borne flavivirus that causes microcephaly and Guillain-Barré syndrome in infected individuals. To obtain insights into the mechanism of ZIKV infection and pathogenesis, we analyzed the transcriptome of ZIKV infected human neural progenitor cells (hNPCs) for changes in alternative splicing (AS), gene isoform (ISO) composition and long noncoding RNAs (lncRNAs) expression.

**Methods:**

We analyzed differentially expressed lncRNAs, AS, ISO from RNA-seq data in ZIKV infected hNPCs.

**Results:**

We obtained 149 differentially expressed lncRNAs, including potential viral targets to modulate cellular processes such as cell cycle, apoptosis and immune response. The infection induced 262 cases of AS occurring in 229 genes, which were enriched in cell death, RNA processing, transport, and neuron development. Among 691 differentially expressed ISOs, upregulated ISOs were enriched in signaling, regulation of transcription, and amino acid biosynthesis, while downregulated ISOs were mostly enriched in cell cycle. Importantly, these analyses revealed specific links between ZIKV induced changes in cellular pathways and the type of changes in the host transcriptome, suggesting important regulatory mechanisms.

**Conclusions:**

Our analyses revealed candidate lncRNAs, AS events and ISOs which may function in ZIKV infection induced cell cycle disruption, apoptosis and attenuation of neurogenesis, and shed light on the roles of lncRNAs, AS and ISOs in virus-host interactions, and would facilitate future studies of ZIKV infection and pathogenesis.

**Electronic supplementary material:**

The online version of this article (10.1186/s12985-017-0882-6) contains supplementary material, which is available to authorized users.

## Background

Zika virus (ZIKV) is a positive-strand RNA virus with a 10,800 nucleotides genome, belonging to the Flaviviridae family and the genus Flavivirus [1]. It includes African and Asian lineages, and could be transmitted by Aedes species mosquitoes, sex, blood transfusion, organ transplantation, and potentially through urine or saliva [2]. Individuals with compromised immunity are particularly susceptible to ZIKV infection and subsequent disease development [3], with symptoms ranging from fever, lethargy, conjunctivitis, rash, and arthralgia [4]. The Guillain-Barré syndrome and congenital microcephaly are two of the well known consequences of ZIKV infection [5]. The recent epidemic of ZIKV infection in South America and parts of Asia has spurred widespread interest in ZIKV infection and pathogenesis, and it is now known that ZIKV infects cells through the entry receptor AXL and causes the activation of Toll-like receptor 3 (TLR3), and consequently, the infection causes attenuated neurogenesis, dysregulated cell cycle, and increased cell apoptosis in hNPCs [6–8]. However, the mechanistic details of ZIKV infection and pathogenesis are mostly unknown.

LncRNAs are defined as transcripts longer than 200 nucleotides, and can be 5^′^ capped and 3′ polyadenylated, but with limited coding potential [9, 10]. LncRNAs participate in diverse processes, ranging from development to diseases [11]. For example, lncRNA MyelOid Rna Regulator of Bim-Induced Death (Morrbid) represses the expression of its neighboring pro-apoptotic gene, Bcl2l11, by recruiting EZH2 to the Bcl2l11 promoter to maintain this gene in a poised state [12]. Nuclear Paraspeckle Assembly Transcript 1 (NEAT1) promotes ATR signaling in response to replication stress and is involved in a negative feedback loop that impairs oncogene-dependent activation of p53 [13]. LncRNAs are also implicated in pathogen-host interactions. For instance, a cellular lncRNA, ncRNA repressor of the nuclear factor of activated T cells (NRON), degrades the Tat protein, and then contributes to HIV-1 latency [14].

Alternative splicing or AS is one of the most important processes in RNA co-transcriptional and post-transcriptional processing. AS events include skipped exon (SE), retained intron (RI), alternative to 5′ splicing site (AS5), alternative to 3′ splicing site (AS3), mutually exclusive exon (MXE), alternative start (altstart), alternative end (altend) and skip multiple exons (SME) [15, 16]. Approximately 94% of human genes are alternatively spliced, producing a much larger number of functional ISOs from a fixed number of genes [15]. AS also participates in a wide range of biological processes including virus-host interactions [17, 18]. Often considered as the consequence of AS, ISOs are mRNAs products that are generated from the same gene loci but are different in their transcription start sites (TSSs), protein coding DNA sequences (CDSs), or untranslated regions (UTRs) [15].

To gain deeper insights into the ZIKV-infected transcriptome, we analyzed RNA-seq data of ZIKV-infected hNPCs [6] to determine the expression of lncRNAs, AS and ISO composition changes, and to predict how these changes are related to the mechanism ZIKV infection and pathogenesis. We identified 149 differentially expressed (DE) lncRNAs in ZIKV-infected samples, and some of these are likely viral targets to modulate cellular processes including cell cycle, apoptosis and immune response. ZIKV infection induced alternatively spliced genes are enriched in cell death, transport, RNA processing, and neuron development, while most of DE ISOs belong to cell cycle, amino acid biosynthesis, transcription, and apoptosis. Our analyses also revealed specific links between ZIKV infection induced changes in cellular pathways and type of changes in the host transcriptome. These results indicated that ZIKV infection induced significant changes in AS, ISOs and lncRNAs expression, which could play key roles in ZIKV infection, virus-host interactions, and pathogenesis.

## Methods

### Prediction of novel lncRNAs

We used FastQC (http://www.bioinformatics.babraham.ac.uk/projects/fastqc/) and FASTX-Toolkit (http://hannonlab.cshl.edu/fastx_toolkit/) to check and filter low quality reads with default parameter. Based on Hu et al. [19], clean reads were mapped to the Human reference genome (Homo_sapiens.GRCh38) using Tophat2 with default parameter [20]. Cufflinks [21] were used to assemble and compare transcripts with reference. Then we extracted candidate novel lncRNAs transcripts with following criteria: the length of transcript ≥ 200 nucleotides, the expression level (fragments per kilobase of exon per million fragments mapped, FPKM) of transcript ≥ 1, the number of exons of trasncript ≥ 2, and class code “i”, “j”, “o”, “u” and “x”. We used CNCI (score < 0) [22] and CPAT (coding probability < 0.364) [23] software to predict the coding potential of discovered lncRNAs.

### LncRNAs and Isoforms differential expression analysis

We used Cuffdiff [24] to analyze expression level of lncRNAs and ISOs, and used the following criteria to select DE lncRNAs and ISOs: FDR ≤ 0.05 and fold-change ≥ 2.

#### Functional prediction of lncRNAs

We used unsupervised machine learning algorithm, k-means, to cluster DE lncRNAs with PCGs.

### Alternative splicing analysis

The bam files generated by Tophat2 [20] were as input files to analyze changes in AS using ASD (v1.2) [25] with the annotation file Homo_sapiens.GRCh38.83.gtf. We selected significant AS cases with an adjusted *p* value ≤ 0.05.

### Gene ontology enrichment and pathway analysis

We uploaded genes with significant ZIKV infection induced PCGs, AS and ISO changes into the Database for Annotation, Visualization and Integrated Discovery (DAVID) v6.7 [26] to perform Gene Ontology functional enrichment analyses (biological processes) and Kyoto Encyclopedia of Genes and Genomes pathway analyses (KEGG). We selected significant GO terms and pathways with a *p* value ≤ 0.05.

### Statistical analysis

We used *R* relative packages, such as pheatmap (pheatmap: Pretty Heatmaps, Raivo Kolde, 2015) and VennDiagram (VennDiagram: Generate High-Resolution Venn and Euler Plots, Hanbo Chen, 2015), and functions, such as Welch Two Sample t-test to analyze data and draw figures.

## Results

### Differentially expressed lncRNAs in ZIKV infected hNPCs

**Fig. 1:**
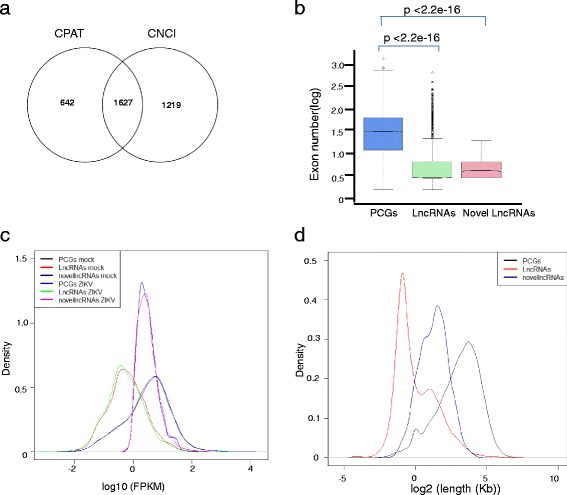
Novel lncRNAs in ZIKV infected human neuro progenitor cells. **a**. The number of candidate novel lncRNAs; **b**. The novel lncRNAs represent smaller the number of exon than PCGs on average; **c**. The PCGs represent higher expression level than novel lncRNAs on average; **d**. The novel lncRNAs show shorter length on average than PCGs .Wilcox.test, *p* value < 0.05

To generate a compendium of all annotated and novel lncRNAs that exhibited differential expression in ZIKV infected hNPCs, we analyzed RNA-seq data generated by using a low multiplicity of infection (MOI < 0.1) of MR766 strain of the ZIKV (African lineage) to infect hNPCs for 56 h [6]. First, we aligned clean RNA-seq data onto human reference genome (Table 1), followed by transcripts assembly and comparison. Using the modified method by Hu et al. [19], we extracted the sequences of candidate novel lncRNAs, and distinguished noncoding RNAs from coding RNAs. As a result, CPAT [23] and CNCI [22] predicted 2269 and 2846 novel lncRNA transcripts, respectively. We discarded 642 lncRNA transcripts predicted by CPAT [23] and 1219 by CNCI [22] with default parameters, respectively, then used 1627 transcripts common to both methods for further analysis (Fig. 1a). Finally, by comparing to the Ensemble database, we obtained 15,275 annotated and 1137 novel lncRNAs, respectively. The novel lncRNAs consist of 1627 transcripts (Fig. 1a).

**Table 1:** The number of reads from RNA-seq data

	mock-1		mock-2		ZIKV-1		ZIKV-2	
Number of reads	Left	Right	Left	Right	Left	Right	Left	Right
Total reads	7,927,777	7,927,777	7,391,076	7,391,076	7,361,527	7,361,527	7,621,347	7,621,347
Mapped reads	7,606,613	7,476,318	7,069,569	6,803,489	7,019,141	6,899,086	7,231,763	7,121,414
Unique reads	7,339,241	7,215,072	6,816,384	6,562,659	6,771,681	6,657,458	6,980,585	6,875,554

“total redas” was the clean reads of RNA-seq data. “mapped reads” was the number of reads aligned to human reference genome. “unique reads” was the number of reads aligned to one site. “Left and Right” was the forward and reverse reads of RNA-seq data, respectively. “mock-1 and mock-2” were control RNA-seq data. “ZIKV-1 and ZIKV-2” were ZIKV infected RNA-seq data

LncRNAs are shorter in length, have fewer exons, and are expressed at much lower levels than protein coding genes (PCGs) [27–31]. To determine if the novel lncRNAs we observed have similar characteristics to the previous studies, we compared these parameters of novel lncRNAs with PCGs, and found that the average exon number in the detected novel lncRNAs was also smaller than that of PCGs (*p* value < 0.05, Welch Two Sample t-test, Fig. 1b). The expression levels of these novel lncRNAs in both control and infected cells were also much lower than that of PCGs (*p* value < 0.05, Welch Two Sample t-test, Fig. 1c and Additional file 1: Figure S1), and the length of novel lncRNAs was much shorter than that of PCGs (*p* value < 0.05, Welch Two Sample t-test) (Fig. 1d and Additional file 1: Figure S1). Thus these novel lncRNAs have the characteristics of annotated lncRNAs.

**Fig. 2:**
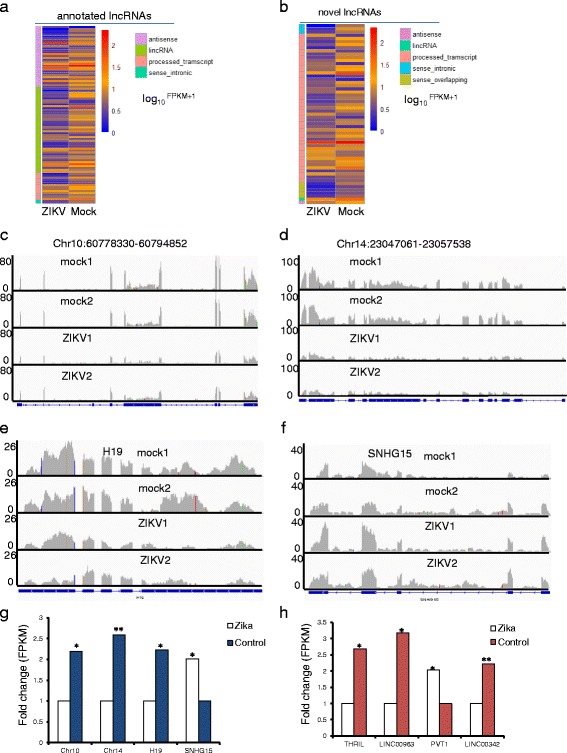
Expression of lncRNAs in ZIKV infected cells. **a**, **b**. heatmap showed 92 annotated and 57 novel DE lncRNAs, respectively. **c**,**d**,**e**,**f** Visualization of two novel and annotated lncRNAs. **g**, **h**. expression level of the indicated lncRNAs. Each group consists of two biological replicates. *: FDR  0.05, **:FDR <0.01

In differentially expressed (DE) lncRNA analysis, we found 92 annotated DE lncRNAs (16 upregulated and 76 downregulated lncRNAs) (Fig. 2a and Additional file 1: Table S1 and Additional file 2) and 57 novel DE lncRNAs (12 upregulated and 45 downregulated lncRNAs) (Fig. 2b and Additional file 1: Table S2 and Additional file 2) in ZIKV infected samples compared to mock samples. These DE lncRNAs represented a diverse group in terms of their biotypes and genomic location. Biotypes included antisense RNA, lincRNA, processed transcript, sense intronic transcript and sense overlapping transcript (Additional file 1: Table S1–2). Two examples of the DE novel lncRNAs, located in Chr10:60,778,330–60,794,852 and Chr14:23,047,061–23,057,538, were downregulated in infected samples (Fig. 2c–g). Likewise, two examples of annotated DE lncRNAs, H19 and SNHG15, are shown in Fig. 2e-f, with the former being downregulated and the latter upregulated by the infection (Fig. 2g).

K-means showed that DE lncRNAs and PCGs might be aggregated into 3 clusters, consisted of 106, 361and 429 genes (lncRNAs and PCGs), respectively (Additional file 1: Table S3 and Figure S2). GO ananlyes showed that downregulated genes from cluster 1 and 2 were mainly enriched in DNA replication and cell cycle. We assumed that downregulated lncRNAs in cluster 1 and 2 might be involved in DNA replication and cell cycle. Upregulated genes from cluster 1 might be involved in amino acid transport and response to stimulation.

From the DE lncRNAs, several stood out as potentially important regulatory factors during ZIKV infection induced immune response and pathogenesis. For example, THRIL (TNFα and hnRNPL related immunoregulatory lincRNA) is known to activate the expression of many immune-response genes, its depletion leads to dysregulation of immune-response genes during innate activation of THP1 macrophages [32]. In the ZIKV infected cells, THRIL, in cluster 2, was downregulated 2.6-fold (Fig. 2h), making it a potential target for ZIKV to modulate host innate immunity.

Apoptosis is an important consequence of ZIKV infection and is believed to be the cause of microcephaly in infected fetus [6]. LINC00963 (long intergenic non coding RNA 963) normally promotes cell survival and plays a role in tumorigenesis [32], its knockdown attenuates C4–2 cell proliferation and induces cell apoptosis [33]. In ZIKV infected hNPC, LINC00963, in cluster 2, was lowered 3-fold (Fig. 2h), making it an important candidate lncRNA mediating ZIKV induced apoptosis.

Cell cycle is dysregulated in ZIKV infected hNPCs [6]. We identified an annotated lincRNA PVT1, Pvt1 oncogene, which was upregulated about 2-fold by the infection and in cluster 1(Fig. 2h). PVT1 knockdown significantly inhibits cell proliferation both in vitro and in vivo, and plays a key role in G1 arrest [34]. Thus, the upregulation of PVT1 could play a role in the disrupted cell cycle due to ZIKV infection. Long intergenic non coding RNA 342, LINC00342 knockdown suppresses proliferation in a lung cancer cell line A549 cells [35]. The downregulation of LINC00342, in cluster 1, in ZIKV infected cells (Fig. 2h) is consistent with its role in ZIKV induced cell cycle effects. Taken together, these analyses suggest that ZIKV infection affected the expression of lncRNAs, and several may serve as targets whereby ZIKV modifies cellular pathways.

Although cluster 3 contained 140 genes, GO analysis showed that these genes were not enriched in any GO terms.

### Changes in alternative splicing after ZIKV infection

**Fig. 3:**
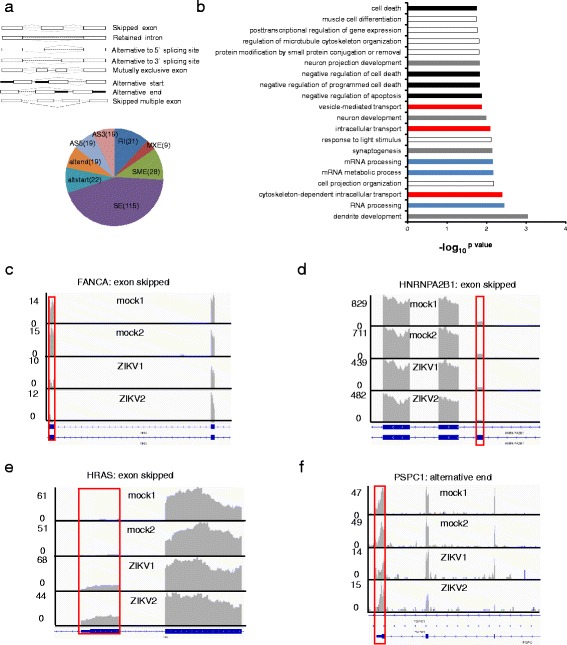
ZIKV infection induced alternative splicing of the cellular transcriptome. **a**. 262 significant differentially AS cases occurred 229 significant alternative splicing genes were detected, which falls into skipped exon, skipped multiple exon, alternative to 5′splicing site (AS5), alternative to 3′splicing site (AS3), alternative start (altstart), alternative end (altend), mutually exclusive and retained intron classes [16]; **b**. GO analysis of AS genes, the x-axis is the –log_10_^p value^, Black, gray, read, and blue denotes cell death, neuron development, transport, and RNA processing, respectively; **c–k**. Visualization of alternative splicing. Y-axis shows the number of mapped reads. **c**. FANCA: increased SE after infection; **d**. HRAS: increased exon after infction; **e**. HNRNPA2B1: reduced exon after infection; **f** PSPC1: reduced altend after infection

Changes in AS have been reported in infected cells by several viruses including HPV, HSV-1 and EBV [16, 36–39]. To determine how host AS was affected by ZIKV infection, we analyzed transcriptome wide AS changes in ZIKV infected hNPCs. AS events (SE, RI, AS5, AS3, MXE, altstart, altend and SME) are graphically illustrated in Fig. 3a [15, 16]. We obtained 262 AS cases occurred in 229 genes (Fig. 3a). While most AS events (200) happened once per gene, they occurred twice in 25 genes, three times in 4 genes, including HMGN1, HNRNPD, MAP2, and PTPRS. The occurrence of different types of AS is summarized in Fig. 3a, among these, the SE type of AS (115 cases) comprised the largest share, of over 43% of all AS events, followed by RI at 12%.

Gene Ontology (GO) functional enrichment analyses of genes undergone AS revealed top 20 GO terms ranked by *p* value, which could be grouped into four broad categories, cell death (4 GO terms), transport (3 GO terms), neuron development (3 GO terms), and RNA processing (3 GO terms) (Fig. 3b and Additional file 1: Table S5-S8). One interesting example, Fanconi anemia complementation group A (FANCA), a DNA repair protein [40], exhibited progressive apoptosis (progressive aplastic anemia) in knockout mice [41]. The 29th exon of FANCA was skipped after ZIKV infection (Fig. 3c), while one of FANCA’s isoforms (id: TCONS_00119591) was downregulated about 2-fold after ZIKV infection (Additional file 1: Figure S4). The function of this exon and this splicing has not been reported and it would be interesting to determine the function of exon 29, and the role of skipping this exon in ZIKV infection.

In the list of RNA processing and transport GO terms, we found Heterogeneous Nuclear Ribonucleoprotein A2/B1 (HNRNPA2B1), involved in pre-mRNA processing [42], underwent AS after ZIKV infection, where the second exon of HNRNPA2B1 was skipped (Fig. 3d). Interestingly, one of HNRNPA2B1’s isoforms (id: TCONS_00285720) was downregulated 2-fold (Additional file 1: Figure S1), while at whole gene level, HNRNPA2B1 was also downregulated about 1.6-fold (*p* value = 0.00035). Thus, the complex changes in HNRNPA2B1 expression, i.e. AS, ISO and DE, suggest its important roles in pre-mRNA processing and transport in infected cells.

H-Ras, a member of the Ras oncogene family [43], was also subject to AS, as its fourth exon was retained after ZIKV infection (Fig. 3e). One of its isoforms (id: TCONS_00049078) was also upregulated about 2.5-fold in infected samples (Additional file 1: Figure S1). As GO analysis suggested that H-Ras might be involved in both cell death and transport, the upregulation of H-Ras could reflect increased apoptosis and nuclear transport as results of the infection.

Interestingly, several genes underwent exclusively AS were enriched in neuron development. For example, Microtubule Associated Protein 2 (MAP2), participating in determining and stabilizing dendritic shape during neuron development [44], was undergone altend type of AS after ZIKV infection (Additional file 1: Table S6 and Figure S4). On the other hand, DCC Netrin 1 Receptor (DCC), which mediates axon attraction of neuronal growth cones in the developing nervous system upon ligand binding [45], was subject to alt3 type of AS following the infection (Additional file 1: Table S6 and Figure S4). The functional consequences of the splicing changes to these genes are unknown, but it would be interesting to determine whether these changes are related to attenuated neurogenesis.

The 3′ UTRs contain multiple miRNA targets [46] and other cis elements [47], allowing fine-tuned regulation of genes. Paraspeckle Component 1 (PSPC1) controls gene expression via an RNA nuclear retention mechanism [48]. We found that there were less mapped reads in the 3′ UTR of PSPC1 in ZIKV infected samples than control samples (Fig. 3f), suggesting PSPC1 would be more stable in infected samples than in mock samples. This might partly explain the extensive alternations in RNA metabolism in infected samples.

### ZIKV infection affected gene isoform composition in the host transcriptome

**Fig. 4:**
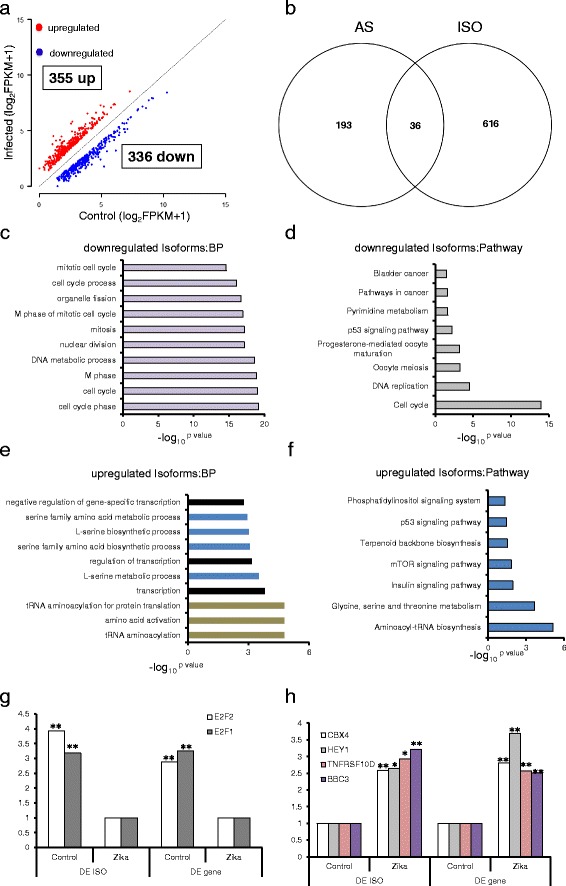
Isoform changes in the ZIKV infected hNPCs transcriptome. **a** A gene with isoform changes of at least 2-fold (FPKM ≥ 1, *FDR* ≤ 0.05) was plotted. A Total of 691 significant isoform changes were found with 355 gene isoforms showing increase of expression and 336 showing reduction. X-axis shows uninfected hNPCs control, and Y-axis shows the amount of isoform expression after infection. Red dots indicate up-regulated gene isoforms and blue dots show reduced isoform expression; **b**. the overlapping genes of AS and ISO; **c**, **d**. GO analysis and pathway analysis of down-regulated isoforms, p value ≤ 0.05; **e**, **f**. GO analysis and pathway analysis of up-regulated isoforms, p value ≤ 0.05. **g**,**h**. expression level of E2F1, E2F2, CXB4, HEY1, TNFRSF10D, and BBC3. Each group consists of two biological replicates. *: FDR < 0.05, **:FDR 0.01. The x-axis of **c**/**d**/**e**/**f** graphs is the –log_**10**_^p value^

To characterize the changes in ISO composition in ZIKV infected samples, we identified 355 cases of increase and 336 cases of decrease in ISOs expression (Fig. 4a and Additional file 1: Table S4). These changes occurred in 652 genes, with 616 genes containing changes in one ISO, 33 genes showing changes in two ISOs, and 3 genes with three ISO changes. When genes with changes in AS and ISO compositon were compared, we found only 36 genes with AS changes also had changes in ISO compositions (Fig. 4b). This is far fewer than expected, as AS is believed to be the main cause of ISO changes, and is likly due to the sequencing depth, as similar results have been observed previously [16, 49, 50].

Interestingly, the top 10 GO terms showed downregulated ISOs were mainly enriched in cell cycle (Fig. 4c), consistent with downregulated DE genes in ZIKV infected samples [6], while pathway analysis found downregulated ISOs were involved in cell cycle, p53 signaling pathway, pathways in cancer, pyrimidine metabolism, and progesterone-mediated oocyte maturation (Fig. 4d). This analysis suggests that the dysregulation of cell cycle process could be a direct result of reduced both cell cycle genes and ISOs expression. Notable examples are E2F Transcription Factor 1 (E2F1) and E2F Transcription Factor 2 (E2F2) from the E2F family of transcription factors, which play crucial roles in the control of cell cycle [51, 52]. E2F1 and E2F2 were downregulated about 3-fold both at gene and ISO levels in infected samples (Fig. 4g).

In contrast, upregulated ISOs were enriched in amino acid biosynthesis and transcription (Fig. 4e), which were also consistent with upregulated DE genes [6]. Upregulated ISOs were also enriched in mTOR signaling pathway, p53 signaling pathway, Insulin signaling pathway, aminoacyl-tRNA biosynthesis, glycine, serine and threonine metabolism, terpenoid backbone, and phosphatidylinositol signaling systembiosynthesis (Fig. 4f). As examples of transcriptional regulation, CBX4, a member of Polycomb group (PcG) multiprotein PRC1-like complex and inhibiting expression of many genes [53–55], and HEY1, a transcriptional repressor binding preferentially to the canonical E box sequence 5-CACGTG-3 [56], were also upregulated both at gene and ISO expression levels for about 2.5 folds after ZIKV infection (Fig. 4h). Two pro-apoptotic factors, TNFRSF10D [57] and BBC3 [58], were also upregulated about 2-fold at both gene and gene ISO levels by ZIKV infection (Fig. 4h). Taken together, ISO analysis suggests that many genes underwent changes at both expression level and ISO compisition, thus gene regulation at the level of ISO composition is as important as gene expression in ZIKV infected cells.

## Discussion

ZIKV infection and pathogenesis are subjects of intense interest, but the mechanisms are still largely unknown. Analysis of host transcriptomic alternations caused by ZIKV infection could identify genes and pathways that function during ZIKV infection and pathogenesis. To obtain a global view of changes in gene splicing, ISO composition and lncRNAs expression in ZIKV infected hNPCs, we analyzed RNA-seq data and identified 15,275 annotated lncRNAs. However, due to low expression levels and potential for fragmentary gene models, we cannot exclude the possibility that novel lncRNAs encode small peptides in our downstream analyses. From these, we obtained 92 significant DE annotated lncRNAs, and 55 significant DE novel lncRNAs in ZIKV infected samples. Additionally, ZIKV infection induced 262 AS events, and 355 upregulated and 336 downregulated ISOs in the ZIKV infected samples.

Most virus infections elicit strong immune response, which is often modulated by the incoming virus to facilitate infection. For example, the nonstructural protein NS5 of ZIKV may attenuate Type I Interferon Signaling by binding and inhibiting STAT2 [9]. Interestingly, THRIL, which normally activates many immune response genes [32], was downregulated in ZIKV infected samples, and could be another viral target to modulated host immunity. As a result, only a few DE PCGs involved in immune response, such as TNFSF13B and RIPK2, were upregulated by ZIKV infection. Consistently, GO analysis of DE PCGs did not obtain any significant immune response GO terms [6].

Apoptosis is highly induced in ZIKV infected cells and may hold the key for diseases including microcephaly. One recent study reported that several apoptotic regulators, including ZMAT3, PMAIP1, TNFRSF10D, BBC3, were upregulated, and cell cycle genes, such as E2F1 and E2F2, were downregulated after ZIKV infection [6]. Importantly, these genes were also similarly regulated at the ISO level, further confirming the importance of these processes in ZIKV infection induced pathogenesis (Additional file 1: Table S4). 17 alternatively spliced genes were also enriched in apoptosis, but they did not show any differential expression at gene and ISO levels (see Additional file 1: Table S5). Interestingly, lncRNAs may also play important roles in apoptosis and cell cycle dysregulation. For example, LINC00963 and LINC00342, anti-apoptotic factors [33, 35], are downregulated by ZIKV infection. Moreover, PVT1, inhibitor of cell proliferation [34], is upregulated by ZIKV infection, and therefore could participate in virus-induced cell cycle dysregulation.

Genes subject to AS are enriched in RNA processing, transport and neuron development, however, several alternatively spliced genes enriched in neuron development did not show any differential expression at gene and ISO levels (Additional file 1: Table S5–7). For instance, MAP2 underwent altend, while DCC was subject to alt3 after ZIKV infection (Additional file 1: Table S6 and Figure S4), even though the expression levels of these two genes remained the same. The mechanism of attenuated neurogenesis in infected individuals is currently unknown. It is possible a direct consequence of cell cycle dysregulation and apoptosis, but splicing changes in neurogenic gene may also play a role and be worthy of further investigation.

In DE ISO analysis, we observed many of the upregulated ISOs were enriched in transcription regulation, amino acid biosynthesis and apoptosis. In contrast, downregulated ISO are mostly concentrated in cell cycle. Interestingly, many genes in this category underwent both DE and ISO and they are mostly regulated similarly by ZIKA infection. For instance, HEY1 and CBX4 were upregulated at both gene epxression and gene ISO levels.

Both lineages (African and Asian strains) efficiently target neural stem cells (NSCs). Neurovirulence and neuropathologies might be related to ZIKV African strain which infects and replicates more efficiently in NSCs and hNPCs than ZIKV Asian strain, and triggers a stronger modulation of cellular homeostasis, including cell cycle progression and anti-viral response [59, 60]. Asian ZIKV strains likely cause chronic infections within the central nervous system (CNS). In addition, there are a lot of differences of the nucleotide and amino acid sequence composition in the genome of African and Asian strains [60], which might indicate MR766 strain specifically alters a limited number of genes expression or AS. Owing to these differences between African and Asian reference strains, we will further compare the changes differences of host transcriptome, such as DE and AS, in African and Asian strains infected samples.

In addition to lncRNA differential expression, changes in AS and ISO composition, we also attempted to analyze changes in alternative polyadenylation (APA), as APA is also an important regulatory mechanism of gene expression [61], however, we did not obtain any significant APA events in ZIKV infected hNPCs. One possibility is that ZIKV infection induces very few, or very low levels of APA. It could also due to the RNA sample preparation method used, where total RNA from infected cells was sequenced, resulting in under representation of 3′ end reads.

## Conclusion

**Fig. 5:**
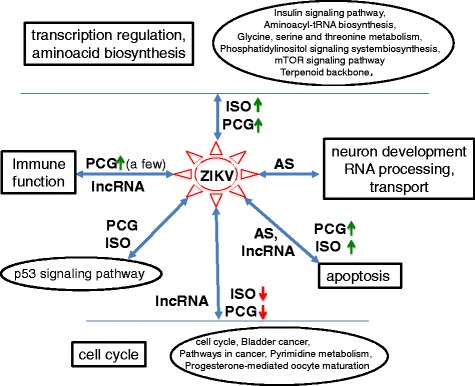
ZIKV induced host responses and alterations of host transcriptome. Several DE lncRNAs, ISOs and PCGs were enriched in cell cycle, apoptosis, immune response, transcription regulation, amino acid biosynthesis, mTOR signaling pathway and p53 signaling pathway. Meanwhile, many of genes undergone AS involved in neuron development, RNA processing and transport. Red and green arrows denote downregulation and upregulation, respectively. The rectangle and oval stand for GO terms and pathways, respectively

Importantly, our analyses suggested that ZIKV infection activated cellular responses or pathways are linked to specific types of changes to the host transcriptome (Fig. 5). For instance, genes enriched in cell cycle, transcription regulation, amino acid biosynthesis, mTOR signaling pathway, and p53 signaling pathway are regulated at the level of differential expression and isoform composition alternations, while neuron development, RNA processing, and transport associated with genes are exclusively regulated at the level of AS after infection (Additional file 1: Table S6-S8). In contrast, genes involved in apoptosis displayed all four types of changes after the infection. These patterns suggest distinct mechanisms of pathway induced transcriptomic changes. This, together with identified lncRNAs and AS events, offers important opportunities to investigate the mechanism of ZIKV infection and pathogenesis.

## Additional files

Additional file 1: Supplemental figures and tables. (DOCX 1754 kb)

Additional file 2: Differential gene expression between Zike Virus infection and control. (XLSX 5758 kb)

## Abbreviations

AS: Alternative splicing
DE: Differentially expressed
GO: Gene Ontology
hNPCs: Human neural progenitor cells
ISO: Gene isoform
lncRNA: Long noncoding RNA
ZIKV: Zika virus
