# Correction to: ZIKV infection effects changes in gene splicing, isoform composition and lncRNA expression in human neural progenitor cells

**DOI:** 10.1186/s12985-019-1122-z

**Published:** 2019-02-04

**Authors:** Benxia Hu, Yongxia Huo, Liping Yang, Guijun Chen, Minhua Luo, Jinlong Yang, Jumin Zhou

**Affiliations:** 1 Key Laboratory of Animal Models and Human Disease Mechanisms of the Chinese Academy of Sciences/Key Laboratory of Bioactive Peptides of Yunnan Province, Kunming Institute of Zoology, Kunming, 650223 China; 2 Kunming College of Life Science, University of Chinese Academy of Sciences, Kunming, 650204 China; 3 State Key Laboratory of Virology, CAS Center for Excellence in Brain Science and Intelligence Technology (CEBSIT), Wuhan Institute of Virology, Wuhan, 430071 China; 4 BGI-Yunnan, BGI-Shenzhen, Kunming, 650000 China; 5 College of Life Sciences, Yunnan University, Kunming, 650091 China

**Correction to: Virol J (2017) 14:217**

**https://doi.org/10.1186/s12985-017-0882-6**

In the original publication of this article [1], two grants from National Science Foundation of China and Yunnan Provincial Government (U1602226) and National Science Foundation of China(2016YFC1200404) were omitted in the ‘Funding’ section. The correct ‘Funding’ section is below.
