# Supplementary material for: ZIKV infection effects changes in gene splicing, isoform composition and lncRNA expression in human neural progenitor cells
Source: Virol J. 2017 Nov 7;14:217. doi: 10.1186/s12985-017-0882-6 (PMC5688814; doi:10.1186/s12985-017-0882-6)
Supplement: Supplementary file 1 — Supplemental figures and tables. (DOCX 1754 kb) [file 12985_2017_882_MOESM1_ESM.docx]

**Table S1.**

| gene | Zika | Control | FDR | type |
| --- | --- | --- | --- | --- |
| RP11-66D17.5 | 2.21692 | 0 | 0.009968 | antisense |
| CTD-2021J15.1 | 0 | 5.24472 | 0.000872 | antisense |
| RP11-146F11.1 | 0.610933 | 5.29423 | 0.018446 | antisense |
| RP11-867G23.8 | 0.438254 | 2.61602 | 0.018446 | antisense |
| RP11-92C4.3 | 1.42426 | 7.60399 | 0.000872 | antisense |
| RP4-591C20.9 | 0.504004 | 2.38254 | 0.004424 | antisense |
| MFI2-AS1 | 1.85246 | 8.37273 | 0.006408 | antisense |
| DLX6-AS1 | 1.28585 | 5.42644 | 0.025266 | antisense |
| RP11-442H21.2 | 104.058 | 24.6684 | 0.000872 | antisense |
| TMEM147-AS1 | 2.466 | 8.22252 | 0.034594 | antisense |
| AC005009.2 | 5.99919 | 1.86094 | 0.031291 | antisense |
| WI2-85898F10.2 | 0.956202 | 3.07925 | 0.02686 | antisense |
| RP11-432J22.2 | 1.34078 | 4.11497 | 0.004942 | antisense |
| RP11-390E23.6 | 1.34293 | 4.05323 | 0.009142 | antisense |
| RP11-379K17.12 | 27.2053 | 9.39256 | 0.003933 | antisense |
| ASB16-AS1 | 2.95726 | 8.07276 | 0.018792 | antisense |
| RP11-23N2.4 | 8.90925 | 3.30367 | 0.000872 | antisense |
| THRIL | 1.59897 | 4.30309 | 0.015724 | antisense |
| GABPB1-AS1 | 5.41759 | 14.4906 | 0.000872 | antisense |
| AC004540.4 | 18.516 | 46.1235 | 0.000872 | antisense |
| LHX5-AS1 | 87.2896 | 215.361 | 0.000872 | antisense |
| RP1-39G22.7 | 4.77001 | 11.2689 | 0.000872 | antisense |
| RP11-498C9.13 | 42.2833 | 18.4931 | 0.048757 | antisense |
| CTA-204B4.2 | 1.28278 | 2.90608 | 0.000872 | antisense |
| SNAI3-AS1 | 2.00865 | 4.54342 | 0.038083 | antisense |
| RP11-573D15.8 | 4.97459 | 2.23732 | 0.027118 | antisense |
| TUG1 | 11.6825 | 25.7222 | 0.014995 | antisense |
| MAFG-AS1 | 2.13433 | 4.48772 | 0.010816 | antisense |
| RP11-649E7.5 | 25.231 | 12.4681 | 0.000872 | antisense |
| LINC01125 | 2.00067 | 5.98994 | 0.000872 | antisense |
| DIO3OS | 4.80892 | 12.0382 | 0.000872 | antisense |
| DGCR5 | 1.75226 | 4.93495 | 0.024964 | lincRNA |
| LINC00894 | 1.55393 | 9.11707 | 0.003394 | lincRNA |
| RP11-191L17.1 | 0 | 1.96244 | 0.010816 | lincRNA |
| CH507-145C22.3 | 1.91157 | 12.7463 | 0.000872 | lincRNA |
| CH507-154B10.1 | 1.91157 | 12.7463 | 0.000872 | lincRNA |
| CH507-154B10.2 | 2.50199 | 14.2899 | 0.009557 | lincRNA |
| LINC00404 | 0.880741 | 4.35584 | 0.020722 | lincRNA |
| AF127936.7 | 4.88326 | 1.31988 | 0.013543 | lincRNA |
| RP11-48B3.4 | 3.96988 | 1.18329 | 0.003933 | lincRNA |
| LINC01560 | 0.746145 | 2.48026 | 0.031016 | lincRNA |
| LINC01021 | 17.5876 | 58.0413 | 0.000872 | lincRNA |
| RP11-492E3.51 | 0.921624 | 2.93122 | 0.042208 | lincRNA |
| RP11-522B15.3 | 2.77964 | 8.51315 | 0.013149 | lincRNA |
| RP11-351J23.1 | 1.61748 | 4.78683 | 0.001592 | lincRNA |
| LINC01239 | 2.02694 | 5.83781 | 0.000872 | lincRNA |
| RP11-725P16.2 | 3.48755 | 9.97562 | 0.000872 | lincRNA |
| ILF3-AS1 | 3.43683 | 9.75898 | 0.000872 | lincRNA |
| KC6 | 4.764 | 13.4201 | 0.000872 | lincRNA |
| DGCR9 | 1.75226 | 4.93495 | 0.024964 | lincRNA |
| LINC01521 | 1.57754 | 4.33681 | 0.000872 | lincRNA |
| PSMG3-AS1 | 1.28394 | 3.50958 | 0.026554 | lincRNA |
| RP11-1055B8.4 | 4.86026 | 13.2622 | 0.000872 | lincRNA |
| RP11-54O7.17 | 2.07757 | 5.6211 | 0.000872 | lincRNA |
| RP11-15H20.7 | 9.76778 | 25.4838 | 0.000872 | lincRNA |
| DKFZP434I0714 | 1.20861 | 3.11855 | 0.006408 | lincRNA |
| AC144652.1 | 4.80732 | 1.90657 | 0.00732 | lincRNA |
| RP11-111M22.3 | 4.99233 | 12.475 | 0.014604 | lincRNA |
| CTC-444N24.8 | 5.50963 | 2.21073 | 0.009142 | lincRNA |
| RP11-1094M14.11 | 3.56485 | 8.88373 | 0.002253 | lincRNA |
| RP11-567M16.6 | 0.931056 | 2.23623 | 0.005445 | lincRNA |
| RP11-395A13.2 | 0.938319 | 2.1867 | 0.002253 | lincRNA |
| RP11-12G12.7 | 0.827604 | 1.90529 | 0.027118 | lincRNA |
| LINC00342 | 10.5395 | 23.4256 | 0.000872 | lincRNA |
| RP11-348J24.2 | 9.15628 | 4.1549 | 0.028127 | lincRNA |
| CH507-254M2.3 | 3.94482 | 8.51528 | 0.03262 | lincRNA |
| CTD-2291D10.4 | 1.38618 | 2.941 | 0.048499 | lincRNA |
| LINC00472 | 3.59934 | 7.56162 | 0.040182 | lincRNA |
| RP3-331H24.5 | 3.59934 | 7.56162 | 0.040182 | lincRNA |
| RP11-307E17.8 | 1.80112 | 3.78207 | 0.008731 | lincRNA |
| PKI55 | 6.281 | 13.1384 | 0.000872 | lincRNA |
| AC159540.1 | 29.3681 | 60.4747 | 0.000872 | lincRNA |
| WI2-85898F10.1 | 6.32505 | 12.9781 | 0.049994 | lincRNA |
| RP11-212P7.2 | 1.68436 | 3.42022 | 0.000872 | lincRNA |
| PVT1 | 11.4513 | 5.64175 | 0.013543 | lincRNA |
| SNHG15 | 20.4323 | 10.1304 | 0.014247 | lincRNA |
| CERS6-AS1 | 0.000957 | 3.98747 | 0.002844 | processed_transcript |
| ITGA9-AS1 | 0.718095 | 2.83091 | 0.009142 | processed_transcript |
| CTD-2228K2.7 | 4.7667 | 16.6315 | 0.000872 | processed_transcript |
| RAD51-AS1 | 1.4245 | 4.83003 | 0.000872 | processed_transcript |
| LINC00963 | 0.921624 | 2.93122 | 0.042208 | processed_transcript |
| RP11-627G23.1 | 0.829494 | 2.41788 | 0.007771 | processed_transcript |
| RP11-15H20.6 | 9.76778 | 25.4838 | 0.000872 | processed_transcript |
| HCG17 | 4.93804 | 12.833 | 0.029634 | processed_transcript |
| HCG18 | 4.93804 | 12.833 | 0.029634 | processed_transcript |
| LAMTOR5-AS1 | 12.6574 | 5.64166 | 0.000872 | processed_transcript |
| H19 | 6.01959 | 13.451 | 0.02365 | processed_transcript |
| CTD-2270P14.1 | 2.30113 | 5.11098 | 0.018446 | processed_transcript |
| RP11-154D6.1 | 3.59934 | 7.56162 | 0.040182 | processed_transcript |
| DANCR | 22.3062 | 45.4185 | 0.000872 | processed_transcript |
| AC000095.11 | 1.75226 | 4.93495 | 0.024964 | sense_intronic |
| AF131215.2 | 2.05542 | 5.42422 | 0.003394 | sense_intronic |

**Table S1. Expression level of DE annotated annotated lncRNAs between ZKV infected and mock infected samples.** Annotated lncRNAs were significant differentially expressed in hNPCs after ZIKV infection. “Zika” and “Control” column denote FPKM value.

**Table S2.**

| locus | Zika | Control | FDR | code |
| --- | --- | --- | --- | --- |
| 3:98902423-99018562 | 0 | 5.24472 | 0.000872 | sense_intronic |
| 7:14145048-14903361 | 1.31067 | 9.7082 | 0.000872 | sense_intronic |
| 18:5954705-6415237 | 1.74941 | 5.73078 | 0.013543 | sense_intronic |
| 2:157736443-157875862 | 10.3957 | 1.53117 | 0.027118 | processed_tramscript |
| 21:6985996-6997819 | 2.50199 | 14.2899 | 0.009557 | processed_tramscript |
| 4:127632931-127726796 | 1.40803 | 7.74138 | 0.000872 | processed_tramscript |
| X:108719948-108738903 | 3.11499 | 14.5979 | 0.000872 | processed_tramscript |
| X:149929526-150224580 | 0.927561 | 4.32315 | 0.032884 | processed_tramscript |
| 15:34851781-34969839 | 8.98199 | 1.95756 | 0.04094 | processed_tramscript |
| 3:196988620-197029816 | 1.85246 | 8.37273 | 0.006408 | processed_tramscript |
| 21:36034540-36126640 | 1.97738 | 7.54497 | 0.037155 | processed_tramscript |
| 12:4538797-4560048 | 3.79575 | 14.221 | 0.000872 | processed_tramscript |
| 21:14818842-15065000 | 4.88326 | 1.31988 | 0.013543 | processed_tramscript |
| 5:473235-524332 | 4.7667 | 16.6315 | 0.000872 | processed_tramscript |
| 16:46689642-46698394 | 4.10114 | 13.9105 | 0.001592 | processed_tramscript |
| 5:27472264-27500095 | 17.5876 | 58.0413 | 0.000872 | processed_tramscript |
| 19:33132091-33176549 | 0.698982 | 2.2712 | 0.024325 | processed_tramscript |
| 10:806699-931705 | 2.42453 | 0.75949 | 0.011144 | processed_tramscript |
| 12:31020762-31104983 | 3.44338 | 10.6673 | 0.045324 | processed_tramscript |
| 15:96354080-96405189 | 2.77964 | 8.51315 | 0.013149 | processed_tramscript |
| 2:118088451-118112321 | 4.13387 | 1.36833 | 0.032388 | processed_tramscript |
| 11:134273528-134319564 | 4.17695 | 12.3845 | 0.000872 | processed_tramscript |
| 6:167678234-167695979 | 1.61748 | 4.78683 | 0.001592 | processed_tramscript |
| 9:22646199-22824213 | 2.02694 | 5.83781 | 0.000872 | processed_tramscript |
| 6:108848415-108974472 | 1.38032 | 3.94475 | 0.015724 | processed_tramscript |
| 8:67952117-68237030 | 2.86868 | 8.14257 | 0.006408 | processed_tramscript |
| 18:41437392-41632185 | 4.764 | 13.4201 | 0.000872 | processed_tramscript |
| 6:30234038-30346884 | 2.40112 | 6.69636 | 0.000872 | processed_tramscript |
| 15:52577841-52679372 | 8.90925 | 3.30367 | 0.000872 | processed_tramscript |
| 7:139231484-139308236 | 3.23787 | 1.21706 | 0.04687 | processed_tramscript |
| 1:53916573-53945929 | 2.59669 | 6.88685 | 0.006872 | processed_tramscript |
| 5:150497777-150558211 | 2.74543 | 7.22376 | 0.03578 | processed_tramscript |
| 12:10699088-10723360 | 3.26373 | 1.26051 | 0.031291 | processed_tramscript |
| 14:23047061-23057538 | 7.43143 | 19.2394 | 0.000872 | processed_tramscript |
| 17:42700192-42745049 | 2.80169 | 7.10172 | 0.042919 | processed_tramscript |
| 22:37051735-37063390 | 5.04458 | 12.5007 | 0.000872 | processed_tramscript |
| 1:153795172-153804022 | 2.38909 | 5.89476 | 0.000872 | processed_tramscript |
| 12:113462033-113480624 | 87.2896 | 215.361 | 0.000872 | processed_tramscript |
| 7:26291894-26376701 | 3.59601 | 8.47659 | 0.03578 | processed_tramscript |
| 3:42547968-42667643 | 20.3797 | 8.98188 | 0.020104 | processed_tramscript |
| 20:3888238-4015586 | 9.70977 | 4.40373 | 0.011144 | processed_tramscript |
| 5:92082596-92688254 | 9.15628 | 4.1549 | 0.028127 | processed_tramscript |
| 22:30922307-30979395 | 11.6825 | 25.7222 | 0.014995 | processed_tramscript |
| 10:60778330-60794852 | 5.10233 | 11.1717 | 0.011885 | processed_tramscript |
| 5:93612751-94111699 | 4.60492 | 10.0781 | 0.000872 | processed_tramscript |
| 6:79201241-79236797 | 17.3963 | 37.8098 | 0.000872 | processed_tramscript |
| 19:23075188-23148928 | 1.38618 | 2.941 | 0.048499 | processed_tramscript |
| 8:127794524-128134239 | 11.4513 | 5.64175 | 0.013543 | processed_tramscript |
| 17:46511510-46579722 | 2.0738 | 4.16566 | 0.023007 | processed_tramscript |
| 15:30616957-30772993 | 1.92827 | 3.87279 | 0.011144 | processed_tramscript |
| 15:96492053-96537039 | 1.03868 | 5.99967 | 0.009968 | sense_overlapping |
| 20:63861006-63865082 | 0.504004 | 2.38254 | 0.004424 | sense_overlapping |
| 12:9398354-9418178 | 1.01474 | 3.47718 | 0.000872 | sense_overlapping |
| 22:45870215-45899223 | 0.956202 | 3.07925 | 0.02686 | sense_overlapping |
| 9:135907811-135916079 | 1.34078 | 4.11497 | 0.004942 | sense_overlapping |
| X:137271783-137377048 | 20.8095 | 41.8448 | 0.000872 | lincRNA |
| 22:45870215-45899223 | 6.32505 | 12.9781 | 0.049994 | antisense |

**Table S2. Expression level of DE novel lncRNAs between ZKV infected and mock infected samples.** Novel lncRNAs were significant differentially expressed in hNPCs after ZIKV infection. “Zika” and “Control” column denote FPKM value.

**Table S3.**

| gene | up/down | type | cluster |
| --- | --- | --- | --- |
| CH507-154B10.2 | down | lncRNA | 1 |
| CTD-2228K2.7 | down | lncRNA | 1 |
| CTD-2228K2.7 | down | lncRNA | 1 |
| DIO3OS | down | lncRNA | 1 |
| GABPB1-AS1 | down | lncRNA | 1 |
| H19 | down | lncRNA | 1 |
| HCG17 | down | lncRNA | 1 |
| HCG18 | down | lncRNA | 1 |
| IGLV1-51 | down | lncRNA | 1 |
| IGLV5-52 | down | lncRNA | 1 |
| KC6 | down | lncRNA | 1 |
| LINC00342 | down | lncRNA | 1 |
| NSUN5P2 | down | lncRNA | 1 |
| PKI55 | down | lncRNA | 1 |
| POLD1,SPIB | down | lncRNA | 1 |
| RP11-1055B8.4 | down | lncRNA | 1 |
| RP11-111M22.3 | down | lncRNA | 1 |
| RP11-15H20.6 | down | lncRNA | 1 |
| RP11-15H20.7 | down | lncRNA | 1 |
| RP11-566E18.1 | down | lncRNA | 1 |
| RP1-39G22.7 | down | lncRNA | 1 |
| TUG1 | down | lncRNA | 1 |
| WI2-85898F10.1 | down | lncRNA | 1 |
| AC006128.2 | up | lncRNA | 1 |
| CCT6P3 | up | lncRNA | 1 |
| LAMTOR5-AS1 | up | lncRNA | 1 |
| LINC00888 | up | lncRNA | 1 |
| PVT1 | up | lncRNA | 1 |
| RN7SK | up | lncRNA | 1 |
| RP11-379K17.12 | up | lncRNA | 1 |
| RP11-460N20.4 | up | lncRNA | 1 |
| RP11-649E7.5 | up | lncRNA | 1 |
| SBDSP1 | up | lncRNA | 1 |
| SNHG15 | up | lncRNA | 1 |
| SNORA9 | up | lncRNA | 1 |
| SEPT10 | down | PCG | 1 |
| ACBD7 | down | PCG | 1 |
| ADHFE1 | down | PCG | 1 |
| AIF1L | down | PCG | 1 |
| ANLN | down | PCG | 1 |
| ARMC6 | down | PCG | 1 |
| ARSB | down | PCG | 1 |
| AS3MT | down | PCG | 1 |
| ASF1B | down | PCG | 1 |
| ASPM | down | PCG | 1 |
| B3GAT3 | down | PCG | 1 |
| BCL2L12 | down | PCG | 1 |
| BIRC5 | down | PCG | 1 |
| BORCS7 | down | PCG | 1 |
| BORCS7-ASMT | down | PCG | 1 |
| BUB1B | down | PCG | 1 |
| C19orf48 | down | PCG | 1 |
| C1orf194 | down | PCG | 1 |
| C21orf58 | down | PCG | 1 |
| C2CD4C | down | PCG | 1 |
| C4orf3 | down | PCG | 1 |
| C7orf49 | down | PCG | 1 |
| C8orf46 | down | PCG | 1 |
| CD82 | down | PCG | 1 |
| CDC45 | down | PCG | 1 |
| CDC7 | down | PCG | 1 |
| CDCA3 | down | PCG | 1 |
| CDCA7 | down | PCG | 1 |
| CDH20 | down | PCG | 1 |
| CDH24 | down | PCG | 1 |
| CDH24 | down | PCG | 1 |
| CDK1 | down | PCG | 1 |
| CDK2 | down | PCG | 1 |
| CDT1 | down | PCG | 1 |
| CENPH | down | PCG | 1 |
| CENPK | down | PCG | 1 |
| CENPM | down | PCG | 1 |
| CENPN | down | PCG | 1 |
| CENPO | down | PCG | 1 |
| CENPU | down | PCG | 1 |
| CHAF1A | down | PCG | 1 |
| CHRAC1 | down | PCG | 1 |
| CKAP2 | down | PCG | 1 |
| CKLF | down | PCG | 1 |
| CKLF-CMTM1 | down | PCG | 1 |
| CMTM1 | down | PCG | 1 |
| CPXM1 | down | PCG | 1 |
| CTC-360G5.8 | down | PCG | 1 |
| CTD-2545M3.6 | down | PCG | 1 |
| CTSF | down | PCG | 1 |
| DBF4B | down | PCG | 1 |
| DCAF16 | down | PCG | 1 |
| DDX11 | down | PCG | 1 |
| DEPDC1B | down | PCG | 1 |
| DLGAP5 | down | PCG | 1 |
| DNPH1 | down | PCG | 1 |
| DPM3 | down | PCG | 1 |
| DTL | down | PCG | 1 |
| E2F1 | down | PCG | 1 |
| EBF2 | down | PCG | 1 |
| EBF4 | down | PCG | 1 |
| EGR2 | down | PCG | 1 |
| ESPL1 | down | PCG | 1 |
| FAM122B | down | PCG | 1 |
| FAM172A | down | PCG | 1 |
| FAM64A | down | PCG | 1 |
| FANCD2 | down | PCG | 1 |
| FBXO17 | down | PCG | 1 |
| FGFBP2 | down | PCG | 1 |
| FGFRL1 | down | PCG | 1 |
| FOXM1 | down | PCG | 1 |
| FZD2 | down | PCG | 1 |
| GDF10 | down | PCG | 1 |
| GFRA1 | down | PCG | 1 |
| GINS1 | down | PCG | 1 |
| GINS2 | down | PCG | 1 |
| GLB1L3 | down | PCG | 1 |
| GMNN | down | PCG | 1 |
| GSTZ1 | down | PCG | 1 |
| HELLS | down | PCG | 1 |
| HMGN3 | down | PCG | 1 |
| IFI27 | down | PCG | 1 |
| IFT81 | down | PCG | 1 |
| IGFBP4 | down | PCG | 1 |
| IMPA2 | down | PCG | 1 |
| ING5 | down | PCG | 1 |
| JRK | down | PCG | 1 |
| KCTD17 | down | PCG | 1 |
| KIAA0141 | down | PCG | 1 |
| KIF11 | down | PCG | 1 |
| KIF20A | down | PCG | 1 |
| KIF23 | down | PCG | 1 |
| KIF4A | down | PCG | 1 |
| KIFC1 | down | PCG | 1 |
| LIG1 | down | PCG | 1 |
| LRRC8A | down | PCG | 1 |
| LTBP1 | down | PCG | 1 |
| LY6E | down | PCG | 1 |
| MAPK11 | down | PCG | 1 |
| MAPK15 | down | PCG | 1 |
| MCM2 | down | PCG | 1 |
| MCM3 | down | PCG | 1 |
| MCM5 | down | PCG | 1 |
| MCM6 | down | PCG | 1 |
| MEGF6 | down | PCG | 1 |
| MFNG | down | PCG | 1 |
| MIS18BP1 | down | PCG | 1 |
| MKI67 | down | PCG | 1 |
| MKRN3 | down | PCG | 1 |
| MMRN1 | down | PCG | 1 |
| MRPL24 | down | PCG | 1 |
| MSH5 | down | PCG | 1 |
| MSH5-SAPCD1 | down | PCG | 1 |
| MYBL2 | down | PCG | 1 |
| MZF1 | down | PCG | 1 |
| NCAPH | down | PCG | 1 |
| NDC1 | down | PCG | 1 |
| NDE1 | down | PCG | 1 |
| NDRG2 | down | PCG | 1 |
| NEMP1 | down | PCG | 1 |
| NME5 | down | PCG | 1 |
| NRBP2 | down | PCG | 1 |
| NUDT1 | down | PCG | 1 |
| OPHN1 | down | PCG | 1 |
| ORC6 | down | PCG | 1 |
| ORC6 | down | PCG | 1 |
| PAK6 | down | PCG | 1 |
| PAQR4 | down | PCG | 1 |
| PBK | down | PCG | 1 |
| PBXIP1 | down | PCG | 1 |
| PCED1A | down | PCG | 1 |
| PDDC1 | down | PCG | 1 |
| PGAM5 | down | PCG | 1 |
| PHKA2 | down | PCG | 1 |
| PIANP | down | PCG | 1 |
| PIGN | down | PCG | 1 |
| PLK4 | down | PCG | 1 |
| POLD3 | down | PCG | 1 |
| POP5 | down | PCG | 1 |
| PPIF | down | PCG | 1 |
| PROM1 | down | PCG | 1 |
| PSRC1 | down | PCG | 1 |
| QTRT1 | down | PCG | 1 |
| RABL2A | down | PCG | 1 |
| RAD51AP1 | down | PCG | 1 |
| RAD51AP1 | down | PCG | 1 |
| RECQL4 | down | PCG | 1 |
| RFC3 | down | PCG | 1 |
| RMI2 | down | PCG | 1 |
| RP6-24A23.6 | down | PCG | 1 |
| RRS1 | down | PCG | 1 |
| SAPCD1 | down | PCG | 1 |
| SARS2 | down | PCG | 1 |
| SDC1 | down | PCG | 1 |
| SDHAF3 | down | PCG | 1 |
| SHMT1 | down | PCG | 1 |
| SLC18A1 | down | PCG | 1 |
| SLC18B1 | down | PCG | 1 |
| SLC19A1 | down | PCG | 1 |
| SMC2 | down | PCG | 1 |
| SOX1 | down | PCG | 1 |
| ST6GALNAC3 | down | PCG | 1 |
| TAGLN | down | PCG | 1 |
| TEX9 | down | PCG | 1 |
| TIMELESS | down | PCG | 1 |
| TK1 | down | PCG | 1 |
| TMEM107 | down | PCG | 1 |
| TMEM168 | down | PCG | 1 |
| TMEM218 | down | PCG | 1 |
| TMEM254 | down | PCG | 1 |
| TRIM45 | down | PCG | 1 |
| TRIM73 | down | PCG | 1 |
| TROAP | down | PCG | 1 |
| TTC14 | down | PCG | 1 |
| TTC31 | down | PCG | 1 |
| TTK | down | PCG | 1 |
| UBTF | down | PCG | 1 |
| UHRF1 | down | PCG | 1 |
| UNC50 | down | PCG | 1 |
| WNT8B | down | PCG | 1 |
| YEATS4 | down | PCG | 1 |
| ZDHHC16 | down | PCG | 1 |
| ZNF519 | down | PCG | 1 |
| ZWINT | down | PCG | 1 |
| ABCA1 | up | PCG | 1 |
| ACBD3 | up | PCG | 1 |
| AGO3 | up | PCG | 1 |
| AHCYL2 | up | PCG | 1 |
| AKAP17A | up | PCG | 1 |
| ALG2 | up | PCG | 1 |
| ALKBH1 | up | PCG | 1 |
| ANKRA2 | up | PCG | 1 |
| ARHGAP23 | up | PCG | 1 |
| ASS1 | up | PCG | 1 |
| ATG2B | up | PCG | 1 |
| BAMBI | up | PCG | 1 |
| C16orf87 | up | PCG | 1 |
| C2CD2L | up | PCG | 1 |
| CAMK2D | up | PCG | 1 |
| CCDC186 | up | PCG | 1 |
| CCDC64 | up | PCG | 1 |
| CEBPB | up | PCG | 1 |
| CHAC1 | up | PCG | 1 |
| CLK4 | up | PCG | 1 |
| COG3 | up | PCG | 1 |
| CREB3L2 | up | PCG | 1 |
| CREBRF | up | PCG | 1 |
| CRY1 | up | PCG | 1 |
| CRY2 | up | PCG | 1 |
| CTH | up | PCG | 1 |
| DGKG | up | PCG | 1 |
| DNAJC1 | up | PCG | 1 |
| DNAJC3 | up | PCG | 1 |
| DUSP14 | up | PCG | 1 |
| EIF2AK3 | up | PCG | 1 |
| ETV5 | up | PCG | 1 |
| FGFR1OP2 | up | PCG | 1 |
| FLCN | up | PCG | 1 |
| GADD45B | up | PCG | 1 |
| GOLGA4 | up | PCG | 1 |
| GOLGA5 | up | PCG | 1 |
| GOLGB1 | up | PCG | 1 |
| GPT2 | up | PCG | 1 |
| IL20RB | up | PCG | 1 |
| IRS2 | up | PCG | 1 |
| JMY | up | PCG | 1 |
| KLF10 | up | PCG | 1 |
| KLHL11 | up | PCG | 1 |
| KLHL15 | up | PCG | 1 |
| KLHL28 | up | PCG | 1 |
| LCORL | up | PCG | 1 |
| LIN7C | up | PCG | 1 |
| LRIF1 | up | PCG | 1 |
| LYSMD3 | up | PCG | 1 |
| MAGEL2 | up | PCG | 1 |
| MBNL2 | up | PCG | 1 |
| MYC | up | PCG | 1 |
| NADK2 | up | PCG | 1 |
| NAMPT | up | PCG | 1 |
| NANS | up | PCG | 1 |
| NCK1 | up | PCG | 1 |
| NEU1 | up | PCG | 1 |
| NFAT5 | up | PCG | 1 |
| NFIL3 | up | PCG | 1 |
| NFXL1 | up | PCG | 1 |
| NKTR | up | PCG | 1 |
| NR4A1 | up | PCG | 1 |
| NUCB2 | up | PCG | 1 |
| OSBP | up | PCG | 1 |
| PCLO | up | PCG | 1 |
| PDGFC | up | PCG | 1 |
| PDXK | up | PCG | 1 |
| PHC3 | up | PCG | 1 |
| PKIB | up | PCG | 1 |
| PLCD1 | up | PCG | 1 |
| PLD6 | up | PCG | 1 |
| PLEKHF2 | up | PCG | 1 |
| PLEKHH3 | up | PCG | 1 |
| PMAIP1 | up | PCG | 1 |
| PPIL4 | up | PCG | 1 |
| PPP2R2B | up | PCG | 1 |
| PSPH | up | PCG | 1 |
| RAB39B | up | PCG | 1 |
| RAD17 | up | PCG | 1 |
| RC3H1 | up | PCG | 1 |
| RILPL1 | up | PCG | 1 |
| RNF169 | up | PCG | 1 |
| RP11-45M22.4 | up | PCG | 1 |
| RYR3 | up | PCG | 1 |
| SAR1B | up | PCG | 1 |
| SEC24A | up | PCG | 1 |
| SESN2 | up | PCG | 1 |
| SLC1A4 | up | PCG | 1 |
| SLC30A1 | up | PCG | 1 |
| SLC39A14 | up | PCG | 1 |
| SLC7A1 | up | PCG | 1 |
| SLC7A11 | up | PCG | 1 |
| SLC7A3 | up | PCG | 1 |
| SNCA | up | PCG | 1 |
| SNX18 | up | PCG | 1 |
| SP8 | up | PCG | 1 |
| SPTY2D1 | up | PCG | 1 |
| SRPRB | up | PCG | 1 |
| SYNJ1 | up | PCG | 1 |
| TBL2 | up | PCG | 1 |
| TGDS | up | PCG | 1 |
| TIMP3 | up | PCG | 1 |
| TIPARP | up | PCG | 1 |
| TMEM214 | up | PCG | 1 |
| TMEM263 | up | PCG | 1 |
| TMEM39A | up | PCG | 1 |
| TRIB3 | up | PCG | 1 |
| TRIM23 | up | PCG | 1 |
| TROVE2 | up | PCG | 1 |
| TRPM4 | up | PCG | 1 |
| TRPS1 | up | PCG | 1 |
| TSC22D3 | up | PCG | 1 |
| TSPYL2 | up | PCG | 1 |
| TVP23B | up | PCG | 1 |
| UBR3 | up | PCG | 1 |
| UHMK1 | up | PCG | 1 |
| UHRF1BP1 | up | PCG | 1 |
| USP15 | up | PCG | 1 |
| UTY | up | PCG | 1 |
| ZBTB43 | up | PCG | 1 |
| ZCCHC8 | up | PCG | 1 |
| ZDBF2 | up | PCG | 1 |
| ZMAT3 | up | PCG | 1 |
| ZNF264 | up | PCG | 1 |
| ZNF433 | up | PCG | 1 |
| ZNF878 | up | PCG | 1 |
| AC000095.11 | down | lncRNA | 2 |
| AC004540.4 | down | lncRNA | 2 |
| AC010980.2 | down | lncRNA | 2 |
| AF131215.2 | down | lncRNA | 2 |
| ANKRD18EP | down | lncRNA | 2 |
| ASB16-AS1 | down | lncRNA | 2 |
| BNIP3P17 | down | lncRNA | 2 |
| CERS6-AS1 | down | lncRNA | 2 |
| CH507-145C22.3 | down | lncRNA | 2 |
| CH507-154B10.1 | down | lncRNA | 2 |
| CH507-254M2.3 | down | lncRNA | 2 |
| CTA-204B4.2 | down | lncRNA | 2 |
| CTC-351M12.1 | down | lncRNA | 2 |
| CTD-2021J15.1 | down | lncRNA | 2 |
| CTD-2270P14.1 | down | lncRNA | 2 |
| CTD-2291D10.4 | down | lncRNA | 2 |
| DDX12P | down | lncRNA | 2 |
| DGCR5 | down | lncRNA | 2 |
| DGCR9 | down | lncRNA | 2 |
| DKFZP434I0714 | down | lncRNA | 2 |
| DLEU2_2 | down | lncRNA | 2 |
| DLX6-AS1 | down | lncRNA | 2 |
| DPY19L2P2 | down | lncRNA | 2 |
| Evf-2_5p | down | lncRNA | 2 |
| FAM27E3 | down | lncRNA | 2 |
| HCG18 | down | lncRNA | 2 |
| ILF3-AS1 | down | lncRNA | 2 |
| IRS4,RP6-24A23.6 | down | lncRNA | 2 |
| ITGA9-AS1 | down | lncRNA | 2 |
| LINC00404 | down | lncRNA | 2 |
| LINC00472 | down | lncRNA | 2 |
| LINC00472 | down | lncRNA | 2 |
| LINC00894 | down | lncRNA | 2 |
| LINC00894 | down | lncRNA | 2 |
| LINC00963 | down | lncRNA | 2 |
| LINC01125 | down | lncRNA | 2 |
| LINC01239 | down | lncRNA | 2 |
| LINC01521 | down | lncRNA | 2 |
| LINC01560 | down | lncRNA | 2 |
| MAFG-AS1 | down | lncRNA | 2 |
| Metazoa_SRP | down | lncRNA | 2 |
| MFI2-AS1 | down | lncRNA | 2 |
| MINCR | down | lncRNA | 2 |
| PSMG3-AS1 | down | lncRNA | 2 |
| RAD51-AS1 | down | lncRNA | 2 |
| RP11-1094M14.11 | down | lncRNA | 2 |
| RP11-12G12.7 | down | lncRNA | 2 |
| RP11-146F11.1 | down | lncRNA | 2 |
| RP11-177G23.1 | down | lncRNA | 2 |
| RP11-191L17.1 | down | lncRNA | 2 |
| RP11-212P7.2 | down | lncRNA | 2 |
| RP11-216N14.5 | down | lncRNA | 2 |
| RP11-307E17.8 | down | lncRNA | 2 |
| RP11-308D16.2 | down | lncRNA | 2 |
| RP11-351J23.1 | down | lncRNA | 2 |
| RP11-390E23.6 | down | lncRNA | 2 |
| RP11-395A13.2 | down | lncRNA | 2 |
| RP11-432J22.2 | down | lncRNA | 2 |
| RP11-492E3.51 | down | lncRNA | 2 |
| RP11-522B15.3 | down | lncRNA | 2 |
| RP11-54O7.17 | down | lncRNA | 2 |
| RP11-567M16.6 | down | lncRNA | 2 |
| RP11-599J14.2 | down | lncRNA | 2 |
| RP11-627G23.1 | down | lncRNA | 2 |
| RP11-65F13.3 | down | lncRNA | 2 |
| RP11-725P16.2 | down | lncRNA | 2 |
| RP11-726G1.1 | down | lncRNA | 2 |
| RP11-867G23.8 | down | lncRNA | 2 |
| RP11-92C4.3 | down | lncRNA | 2 |
| RP3-331H24.5 | down | lncRNA | 2 |
| RP4-591C20.9 | down | lncRNA | 2 |
| RPS27P25 | down | lncRNA | 2 |
| Six3os1_1 | down | lncRNA | 2 |
| SNAI3-AS1 | down | lncRNA | 2 |
| SNRPGP10 | down | lncRNA | 2 |
| THRIL | down | lncRNA | 2 |
| TMEM147-AS1 | down | lncRNA | 2 |
| WI2-85898F10.2 | down | lncRNA | 2 |
| AC005009.2 | up | lncRNA | 2 |
| AC009963.3 | up | lncRNA | 2 |
| AC016717.1 | up | lncRNA | 2 |
| AC144652.1 | up | lncRNA | 2 |
| AF127936.7 | up | lncRNA | 2 |
| CCT6P1 | up | lncRNA | 2 |
| CH17-13I23.3 | up | lncRNA | 2 |
| CTC-444N24.8 | up | lncRNA | 2 |
| HSD17B7P2 | up | lncRNA | 2 |
| MIR4492 | up | lncRNA | 2 |
| RN7SL292P | up | lncRNA | 2 |
| RNA5SP37 | up | lncRNA | 2 |
| RNU6-216P | up | lncRNA | 2 |
| RNU6-322P | up | lncRNA | 2 |
| RP11-23N2.4 | up | lncRNA | 2 |
| RP11-326C3.16 | up | lncRNA | 2 |
| RP11-348J24.2 | up | lncRNA | 2 |
| RP11-48B3.4 | up | lncRNA | 2 |
| RP11-573D15.8 | up | lncRNA | 2 |
| RP11-66D17.5 | up | lncRNA | 2 |
| SMG1P5 | up | lncRNA | 2 |
| Z69720.3 | up | lncRNA | 2 |
| ABCC4 | down | PCG | 2 |
| ACTC1 | down | PCG | 2 |
| ADAM11 | down | PCG | 2 |
| ADAMTS15 | down | PCG | 2 |
| ANKRD34B | down | PCG | 2 |
| ARHGAP11B | down | PCG | 2 |
| ARHGEF39 | down | PCG | 2 |
| ARHGEF6 | down | PCG | 2 |
| ARL17A | down | PCG | 2 |
| ARMC2 | down | PCG | 2 |
| ARMC3 | down | PCG | 2 |
| ATAD5 | down | PCG | 2 |
| ATG16L2 | down | PCG | 2 |
| ATHL1 | down | PCG | 2 |
| BHLHE23 | down | PCG | 2 |
| BOK | down | PCG | 2 |
| BRI3BP | down | PCG | 2 |
| BRIP1 | down | PCG | 2 |
| C18orf54 | down | PCG | 2 |
| C1GALT1C1 | down | PCG | 2 |
| C1orf106 | down | PCG | 2 |
| C1orf216 | down | PCG | 2 |
| C1QTNF6 | down | PCG | 2 |
| C1R | down | PCG | 2 |
| C1RL | down | PCG | 2 |
| C5 | down | PCG | 2 |
| C6orf118 | down | PCG | 2 |
| C8orf44 | down | PCG | 2 |
| C8orf44-SGK3 | down | PCG | 2 |
| CASC10 | down | PCG | 2 |
| CCDC173 | down | PCG | 2 |
| CCNO | down | PCG | 2 |
| CD248 | down | PCG | 2 |
| CD9 | down | PCG | 2 |
| CDC20B | down | PCG | 2 |
| CDKN2C | down | PCG | 2 |
| CEP152 | down | PCG | 2 |
| CFAP52 | down | PCG | 2 |
| CHRNA1 | down | PCG | 2 |
| CILP2 | down | PCG | 2 |
| CLEC11A | down | PCG | 2 |
| CLSPN | down | PCG | 2 |
| COBL | down | PCG | 2 |
| CREG1 | down | PCG | 2 |
| CSGALNACT1 | down | PCG | 2 |
| CTSC | down | PCG | 2 |
| CYP26B1 | down | PCG | 2 |
| CYP27C1 | down | PCG | 2 |
| DARS2 | down | PCG | 2 |
| DBX1 | down | PCG | 2 |
| DDX11 | down | PCG | 2 |
| DEPDC1 | down | PCG | 2 |
| DGKB | down | PCG | 2 |
| DHRS11 | down | PCG | 2 |
| DNA2 | down | PCG | 2 |
| DNAI1 | down | PCG | 2 |
| DPY19L2 | down | PCG | 2 |
| DRP2 | down | PCG | 2 |
| DSCC1 | down | PCG | 2 |
| DUSP23 | down | PCG | 2 |
| E2F2 | down | PCG | 2 |
| E2F3 | down | PCG | 2 |
| EFCAB12 | down | PCG | 2 |
| EFEMP1 | down | PCG | 2 |
| ELOVL2 | down | PCG | 2 |
| EMB | down | PCG | 2 |
| EMID1 | down | PCG | 2 |
| EMILIN3 | down | PCG | 2 |
| EMP2 | down | PCG | 2 |
| ERI1 | down | PCG | 2 |
| EZH1 | down | PCG | 2 |
| FAM107A | down | PCG | 2 |
| FAM111B | down | PCG | 2 |
| FANK1 | down | PCG | 2 |
| FBLN7 | down | PCG | 2 |
| FGF9 | down | PCG | 2 |
| FOXN4 | down | PCG | 2 |
| GALNT12 | down | PCG | 2 |
| GDPD2 | down | PCG | 2 |
| GEN1 | down | PCG | 2 |
| GINS3 | down | PCG | 2 |
| GLB1L | down | PCG | 2 |
| GM2A | down | PCG | 2 |
| GPSM3 | down | PCG | 2 |
| GRIN2A | down | PCG | 2 |
| HSD17B8 | down | PCG | 2 |
| HSPB11 | down | PCG | 2 |
| IGFBP5 | down | PCG | 2 |
| IL13RA1 | down | PCG | 2 |
| INTU | down | PCG | 2 |
| IQGAP3 | down | PCG | 2 |
| KANK2 | down | PCG | 2 |
| KDELC2 | down | PCG | 2 |
| KIF19 | down | PCG | 2 |
| KIF9 | down | PCG | 2 |
| KLHDC9 | down | PCG | 2 |
| KLRG1 | down | PCG | 2 |
| L3MBTL4 | down | PCG | 2 |
| LPL | down | PCG | 2 |
| LUM | down | PCG | 2 |
| MACC1 | down | PCG | 2 |
| MCM10 | down | PCG | 2 |
| MED11 | down | PCG | 2 |
| MMP2 | down | PCG | 2 |
| MMS22L | down | PCG | 2 |
| MURC | down | PCG | 2 |
| MYLK | down | PCG | 2 |
| NDST1 | down | PCG | 2 |
| NID1 | down | PCG | 2 |
| NOTCH4 | down | PCG | 2 |
| NRGN | down | PCG | 2 |
| NTSR1 | down | PCG | 2 |
| OAF | down | PCG | 2 |
| OLFML2B | down | PCG | 2 |
| OLIG3 | down | PCG | 2 |
| OPCML | down | PCG | 2 |
| ORC1 | down | PCG | 2 |
| PAQR6 | down | PCG | 2 |
| PDLIM1 | down | PCG | 2 |
| PEX11A | down | PCG | 2 |
| PGAP3 | down | PCG | 2 |
| PIF1 | down | PCG | 2 |
| PLCE1 | down | PCG | 2 |
| PLD2 | down | PCG | 2 |
| PLEKHB1 | down | PCG | 2 |
| PLEKHM1 | down | PCG | 2 |
| PNMA3 | down | PCG | 2 |
| POLA2 | down | PCG | 2 |
| PREX2 | down | PCG | 2 |
| PROCA1 | down | PCG | 2 |
| PTCHD4 | down | PCG | 2 |
| PTGFRN | down | PCG | 2 |
| PTPN18 | down | PCG | 2 |
| PUS7 | down | PCG | 2 |
| PYCRL | down | PCG | 2 |
| RAD51 | down | PCG | 2 |
| RASSF7 | down | PCG | 2 |
| RBL1 | down | PCG | 2 |
| RBM41 | down | PCG | 2 |
| RP11-403P17.5 | down | PCG | 2 |
| RRM2 | down | PCG | 2 |
| SAPCD2 | down | PCG | 2 |
| SCARA3 | down | PCG | 2 |
| SDC4 | down | PCG | 2 |
| SETD4 | down | PCG | 2 |
| SFXN2 | down | PCG | 2 |
| SGK3 | down | PCG | 2 |
| SGOL2 | down | PCG | 2 |
| SIM1 | down | PCG | 2 |
| SIRPA | down | PCG | 2 |
| SLC35G1 | down | PCG | 2 |
| SLC44A3 | down | PCG | 2 |
| SLC4A4 | down | PCG | 2 |
| SLC6A16 | down | PCG | 2 |
| SPAG17 | down | PCG | 2 |
| SPAG6 | down | PCG | 2 |
| SPAG8 | down | PCG | 2 |
| SPARCL1 | down | PCG | 2 |
| SPOCK1 | down | PCG | 2 |
| SSPO | down | PCG | 2 |
| STXBP6 | down | PCG | 2 |
| SUSD2 | down | PCG | 2 |
| TAS2R5 | down | PCG | 2 |
| TCF19 | down | PCG | 2 |
| TK2 | down | PCG | 2 |
| TKTL1 | down | PCG | 2 |
| TLN2 | down | PCG | 2 |
| TMEM17 | down | PCG | 2 |
| TMSB4Y | down | PCG | 2 |
| TNFAIP8 | down | PCG | 2 |
| TNFRSF13C | down | PCG | 2 |
| TRAF5 | down | PCG | 2 |
| TRIL | down | PCG | 2 |
| TRMO | down | PCG | 2 |
| TRPC3 | down | PCG | 2 |
| TSPAN19 | down | PCG | 2 |
| TTF2 | down | PCG | 2 |
| UQCRHL | down | PCG | 2 |
| USP43 | down | PCG | 2 |
| VWA1 | down | PCG | 2 |
| WDR76 | down | PCG | 2 |
| WDR88 | down | PCG | 2 |
| XRCC2 | down | PCG | 2 |
| ACRC | up | PCG | 2 |
| ACSL6 | up | PCG | 2 |
| ACVR1 | up | PCG | 2 |
| ADRA1A | up | PCG | 2 |
| ALDH1L2 | up | PCG | 2 |
| AMMECR1 | up | PCG | 2 |
| ANKRD33B | up | PCG | 2 |
| AQR | up | PCG | 2 |
| ARHGAP27 | up | PCG | 2 |
| ARHGAP9 | up | PCG | 2 |
| ARL14EPL | up | PCG | 2 |
| ATXN1 | up | PCG | 2 |
| BHLHE40 | up | PCG | 2 |
| BMP2 | up | PCG | 2 |
| BORCS5 | up | PCG | 2 |
| C10orf11 | up | PCG | 2 |
| C14orf28 | up | PCG | 2 |
| CAMKMT | up | PCG | 2 |
| CCDC81 | up | PCG | 2 |
| CDC42EP1 | up | PCG | 2 |
| COQ10B | up | PCG | 2 |
| CPEB3 | up | PCG | 2 |
| CRACR2A | up | PCG | 2 |
| CREB3L1 | up | PCG | 2 |
| CSRNP1 | up | PCG | 2 |
| CTB-127M13.1 | up | PCG | 2 |
| CTD-2287O16.3 | up | PCG | 2 |
| DDR2 | up | PCG | 2 |
| DDX58 | up | PCG | 2 |
| DIRAS2 | up | PCG | 2 |
| EEF2K | up | PCG | 2 |
| ELL | up | PCG | 2 |
| ELMSAN1 | up | PCG | 2 |
| EPHX1 | up | PCG | 2 |
| EXOC8 | up | PCG | 2 |
| FAM222A | up | PCG | 2 |
| FAS | up | PCG | 2 |
| FOSL1 | up | PCG | 2 |
| FOXO1 | up | PCG | 2 |
| FRMD3 | up | PCG | 2 |
| FUT3 | up | PCG | 2 |
| GAN | up | PCG | 2 |
| GDF15 | up | PCG | 2 |
| GFOD1 | up | PCG | 2 |
| HBS1L | up | PCG | 2 |
| HCFC2 | up | PCG | 2 |
| HEY1 | up | PCG | 2 |
| HSD17B14 | up | PCG | 2 |
| INHBE | up | PCG | 2 |
| INO80D | up | PCG | 2 |
| INSIG2 | up | PCG | 2 |
| JDP2 | up | PCG | 2 |
| KCNA3 | up | PCG | 2 |
| KCTD16 | up | PCG | 2 |
| KLF15 | up | PCG | 2 |
| LARP4B | up | PCG | 2 |
| LVRN | up | PCG | 2 |
| MAFB | up | PCG | 2 |
| MAP7D3 | up | PCG | 2 |
| MEF2C | up | PCG | 2 |
| MEIKIN | up | PCG | 2 |
| NCOA7 | up | PCG | 2 |
| NFIA | up | PCG | 2 |
| NFKB2 | up | PCG | 2 |
| NFKBIE | up | PCG | 2 |
| NKAIN2 | up | PCG | 2 |
| NOG | up | PCG | 2 |
| NR4A3 | up | PCG | 2 |
| OSBPL6 | up | PCG | 2 |
| OTUD1 | up | PCG | 2 |
| P3H2 | up | PCG | 2 |
| PCDH20 | up | PCG | 2 |
| PCK2 | up | PCG | 2 |
| PDP2 | up | PCG | 2 |
| PGF | up | PCG | 2 |
| PGPEP1 | up | PCG | 2 |
| PHLDB3 | up | PCG | 2 |
| PID1 | up | PCG | 2 |
| PLPPR4 | up | PCG | 2 |
| PPP1R3B | up | PCG | 2 |
| PPP1R3C | up | PCG | 2 |
| PPP3CC | up | PCG | 2 |
| PURA | up | PCG | 2 |
| RELB | up | PCG | 2 |
| RENBP | up | PCG | 2 |
| RGPD5 | up | PCG | 2 |
| RND1 | up | PCG | 2 |
| RNF113A | up | PCG | 2 |
| RNF24 | up | PCG | 2 |
| RORA | up | PCG | 2 |
| RP11-310K10.1 | up | PCG | 2 |
| RRBP1 | up | PCG | 2 |
| SAMD4A | up | PCG | 2 |
| SEC22A | up | PCG | 2 |
| SEC24D | up | PCG | 2 |
| SEMA4A | up | PCG | 2 |
| SERTAD1 | up | PCG | 2 |
| SHISA2 | up | PCG | 2 |
| SIAH2 | up | PCG | 2 |
| SIX4 | up | PCG | 2 |
| SLC10A7 | up | PCG | 2 |
| SOCS2 | up | PCG | 2 |
| STC2 | up | PCG | 2 |
| STK10 | up | PCG | 2 |
| SYDE2 | up | PCG | 2 |
| TNFRSF10D | up | PCG | 2 |
| TUFT1 | up | PCG | 2 |
| TYW5 | up | PCG | 2 |
| UBN2 | up | PCG | 2 |
| ULBP1 | up | PCG | 2 |
| VCPIP1 | up | PCG | 2 |
| WHAMM | up | PCG | 2 |
| YBX3 | up | PCG | 2 |
| ZBTB49 | up | PCG | 2 |
| ZBTB7B | up | PCG | 2 |
| ZMAT4 | up | PCG | 2 |
| ZNF442 | up | PCG | 2 |
| ZNF525 | up | PCG | 2 |
| ZNF543 | up | PCG | 2 |
| ZNF555 | up | PCG | 2 |
| ZNF79 | up | PCG | 2 |
| ZNF805 | up | PCG | 2 |
| ZNF844 | up | PCG | 2 |
| ZXDB | up | PCG | 2 |
| Novel lncRNA | down | lncRNA | 3 |
| AC004540.4 | down | lncRNA | 3 |
| AC159540.1 | down | lncRNA | 3 |
| DANCR | down | lncRNA | 3 |
| LHX5-AS1 | down | lncRNA | 3 |
| LINC01021 | down | lncRNA | 3 |
| AC245033.1 | up | lncRNA | 3 |
| GOLGA2P10 | up | lncRNA | 3 |
| RP11-442H21.2 | up | lncRNA | 3 |
| RP11-498C9.13 | up | lncRNA | 3 |
| AKT2 | down | PCG | 3 |
| ANKUB1 | down | PCG | 3 |
| ARGLU1 | down | PCG | 3 |
| ATF6B | down | PCG | 3 |
| AURKB | down | PCG | 3 |
| BCL2L2 | down | PCG | 3 |
| BCL2L2-PABPN1 | down | PCG | 3 |
| BTBD17 | down | PCG | 3 |
| C14orf37 | down | PCG | 3 |
| CCNB1 | down | PCG | 3 |
| CCNB2 | down | PCG | 3 |
| CDK1 | down | PCG | 3 |
| CHRNA3 | down | PCG | 3 |
| CKS1B | down | PCG | 3 |
| CSRP2 | down | PCG | 3 |
| CTGF | down | PCG | 3 |
| DHFR | down | PCG | 3 |
| DKC1 | down | PCG | 3 |
| DLL3 | down | PCG | 3 |
| EGR1 | down | PCG | 3 |
| EXTL2 | down | PCG | 3 |
| FGFBP3 | down | PCG | 3 |
| FOS | down | PCG | 3 |
| FOSB | down | PCG | 3 |
| GJA1 | down | PCG | 3 |
| GPC3 | down | PCG | 3 |
| H2AFX | down | PCG | 3 |
| HES4 | down | PCG | 3 |
| HES6 | down | PCG | 3 |
| HMGN3 | down | PCG | 3 |
| KLHDC8A | down | PCG | 3 |
| LENG8 | down | PCG | 3 |
| LMNB1 | down | PCG | 3 |
| LUC7L3 | down | PCG | 3 |
| MAT2A | down | PCG | 3 |
| MCM4 | down | PCG | 3 |
| METTL3 | down | PCG | 3 |
| MFAP2 | down | PCG | 3 |
| MFGE8 | down | PCG | 3 |
| NCAPD2 | down | PCG | 3 |
| NES | down | PCG | 3 |
| NUSAP1 | down | PCG | 3 |
| PABPN1 | down | PCG | 3 |
| PAICS | down | PCG | 3 |
| PCNA | down | PCG | 3 |
| PFN2 | down | PCG | 3 |
| PTN | down | PCG | 3 |
| RBBP7 | down | PCG | 3 |
| RRM1 | down | PCG | 3 |
| SECISBP2 | down | PCG | 3 |
| SFRP2 | down | PCG | 3 |
| SMC4 | down | PCG | 3 |
| SNRPA | down | PCG | 3 |
| SRSF5 | down | PCG | 3 |
| TNXB | down | PCG | 3 |
| TOP2A | down | PCG | 3 |
| TPBG | down | PCG | 3 |
| TUBA1B | down | PCG | 3 |
| TUBB4B | down | PCG | 3 |
| TYMS | down | PCG | 3 |
| UBE2C | down | PCG | 3 |
| WNT7B | down | PCG | 3 |
| WSB1 | down | PCG | 3 |
| AARS | up | PCG | 3 |
| ACSL3 | up | PCG | 3 |
| AKAP8L | up | PCG | 3 |
| ARHGAP5 | up | PCG | 3 |
| ASNS | up | PCG | 3 |
| ATXN2 | up | PCG | 3 |
| BBC3 | up | PCG | 3 |
| CA2 | up | PCG | 3 |
| CAGE1 | up | PCG | 3 |
| CARS | up | PCG | 3 |
| CBARP | up | PCG | 3 |
| CBX4 | up | PCG | 3 |
| CDK2AP2 | up | PCG | 3 |
| CDKN2AIP | up | PCG | 3 |
| CEP78 | up | PCG | 3 |
| CUL3 | up | PCG | 3 |
| DDIT3 | up | PCG | 3 |
| DDIT4 | up | PCG | 3 |
| DDX3Y | up | PCG | 3 |
| DNAJB11 | up | PCG | 3 |
| DNAJB9 | up | PCG | 3 |
| EIF4EBP1 | up | PCG | 3 |
| GARS | up | PCG | 3 |
| HERPUD1 | up | PCG | 3 |
| HM13 | up | PCG | 3 |
| HSPA9 | up | PCG | 3 |
| HYOU1 | up | PCG | 3 |
| IARS | up | PCG | 3 |
| INSIG1 | up | PCG | 3 |
| JMJD1C | up | PCG | 3 |
| LMO4 | up | PCG | 3 |
| MARS | up | PCG | 3 |
| MTHFD2 | up | PCG | 3 |
| MYADML2 | up | PCG | 3 |
| NEK7 | up | PCG | 3 |
| NFE2L2 | up | PCG | 3 |
| NUDT4 | up | PCG | 3 |
| PHGDH | up | PCG | 3 |
| PLK2 | up | PCG | 3 |
| POLR2A | up | PCG | 3 |
| PPP1R15B | up | PCG | 3 |
| PYCR1 | up | PCG | 3 |
| RAB5A | up | PCG | 3 |
| RIOK3 | up | PCG | 3 |
| SAFB2 | up | PCG | 3 |
| SARS | up | PCG | 3 |
| SEC23B | up | PCG | 3 |
| SHMT2 | up | PCG | 3 |
| SLC1A5 | up | PCG | 3 |
| SLC3A2 | up | PCG | 3 |
| SLC7A5 | up | PCG | 3 |
| SMIM14 | up | PCG | 3 |
| SND1 | up | PCG | 3 |
| SSR1 | up | PCG | 3 |
| SSR3 | up | PCG | 3 |
| SUGT1 | up | PCG | 3 |
| TAF1D | up | PCG | 3 |
| TARS | up | PCG | 3 |
| TIMM44 | up | PCG | 3 |
| TUBE1 | up | PCG | 3 |
| UBE2B | up | PCG | 3 |
| UGDH | up | PCG | 3 |
| VEGFA | up | PCG | 3 |
| VIMP | up | PCG | 3 |
| XBP1 | up | PCG | 3 |
| XPOT | up | PCG | 3 |
| YARS | up | PCG | 3 |

**Table S3. Cluster of DE lncRNAs and PCGs between ZKV infected and mock infected samples.** “Type” column denotes cluster.

**Table S4.**

| isoform | Zika | Control | FDR | up/down |
| --- | --- | --- | --- | --- |
| ZXDC | 7.85228 | 1.90418 | 0.048958 | up |
| ZXDB | 7.5842 | 3.30292 | 0.004024 | up |
| ZNF844 | 7.10683 | 2.27826 | 0.004024 | up |
| ZNF805 | 5.8511 | 2.33648 | 0.004024 | up |
| ZNF74 | 4.98924 | 0.74215 | 0.026025 | up |
| ZNF625-ZNF20 | 3.48977 | 1.56808 | 0.022555 | up |
| ZNF609 | 6.4527 | 2.10857 | 0.04218 | up |
| ZNF518B | 9.24282 | 4.25931 | 0.004024 | up |
| ZNF316 | 8.55381 | 2.75854 | 0.012156 | up |
| ZNF264 | 7.85992 | 3.19325 | 0.020597 | up |
| ZNF253 | 9.75951 | 4.16295 | 0.004024 | up |
| ZMAT3 | 10.2301 | 3.45931 | 0.022555 | up |
| ZC3H11A | 9.82175 | 4.65968 | 0.04218 | up |
| ZBTB34 | 9.99049 | 4.60351 | 0.027421 | up |
| ZBTB11 | 3.08367 | 0.869031 | 0.027421 | up |
| YTHDC2 | 6.68541 | 3.2293 | 0.033564 | up |
| YTHDC1 | 12.5112 | 6.10094 | 0.009746 | up |
| YOD1 | 18.5704 | 9.179 | 0.004024 | up |
| YARS | 64.9135 | 20.6634 | 0.012156 | up |
| YAF2 | 15.4926 | 7.66417 | 0.026025 | up |
| XPOT | 36.0356 | 8.17257 | 0.004024 | up |
| XPNPEP3 | 3.9091 | 1.08485 | 0.004024 | up |
| XBP1 | 69.829 | 17.9467 | 0.004024 | up |
| WHAMM | 4.36389 | 1.32038 | 0.004024 | up |
| VWA5A | 3.71631 | 0.580761 | 0.037864 | up |
| VIMP | 46.0953 | 19.2109 | 0.004024 | up |
| VCPIP1 | 6.37249 | 2.9141 | 0.004024 | up |
| UTP6 | 6.01123 | 2.30647 | 0.035058 | up |
| USP32 | 6.98177 | 2.76782 | 0.024156 | up |
| USP25 | 6.03388 | 3.0045 | 0.040714 | up |
| USP15 | 12.7292 | 6.31487 | 0.0186 | up |
| UHRF1BP1L | 5.98521 | 2.8232 | 0.016692 | up |
| UHRF1BP1 | 9.42976 | 3.58116 | 0.004024 | up |
| UFM1 | 11.5955 | 5.42098 | 0.0186 | up |
| UBR3 | 12.6343 | 5.47615 | 0.004024 | up |
| U2SURP | 8.38549 | 3.56974 | 0.027421 | up |
| TUFT1 | 6.28029 | 2.29101 | 0.004024 | up |
| TUBE1 | 8.95009 | 2.13379 | 0.035058 | up |
| TTBK2 | 7.71069 | 2.48765 | 0.022555 | up |
| TSC22D3 | 17.9222 | 5.90398 | 0.022555 | up |
| TSC22D2 | 9.40908 | 3.46588 | 0.004024 | up |
| TSC22D2 | 22.8067 | 10.7159 | 0.029065 | up |
| TRPS1 | 4.66413 | 1.82212 | 0.039327 | up |
| TROVE2 | 13.9349 | 6.52243 | 0.004024 | up |
| TRIO | 9.92716 | 4.77317 | 0.029065 | up |
| TRIM23 | 13.4284 | 5.01448 | 0.007147 | up |
| TRIM11 | 8.81046 | 4.234 | 0.007147 | up |
| TRIB3 | 17.0996 | 2.1542 | 0.004024 | up |
| TRAM1 | 69.3205 | 32.4667 | 0.004024 | up |
| TRA2A | 54.3295 | 18.3549 | 0.007147 | up |
| TRA2A | 31.187 | 12.2005 | 0.004024 | up |
| TNRC6B | 3.95945 | 1.6421 | 0.016692 | up |
| TNKS1BP1 | 5.27789 | 0.789192 | 0.033564 | up |
| TNFRSF10D | 2.34413 | 0.799535 | 0.037864 | up |
| TMEM263 | 15.5105 | 5.88644 | 0.004024 | up |
| TMEM214 | 14.7219 | 5.40862 | 0.004024 | up |
| TMEM200A | 11.2625 | 4.84246 | 0.029065 | up |
| TMEM106B | 7.7456 | 2.24879 | 0.004024 | up |
| TMED7-TICAM2 | 26.3362 | 7.37822 | 0.004024 | up |
| TIMP3 | 15.7511 | 6.8716 | 0.004024 | up |
| TIGD2 | 4.45258 | 2.10482 | 0.046221 | up |
| TGDS | 10.8327 | 4.30184 | 0.004024 | up |
| TET3 | 8.03007 | 1.68652 | 0.009746 | up |
| TET2 | 3.68545 | 0.77252 | 0.004024 | up |
| TEF | 6.53526 | 3.04755 | 0.004024 | up |
| TCEB3 | 10.0108 | 3.99902 | 0.004024 | up |
| TARS | 29.902 | 5.1498 | 0.004024 | up |
| TARS | 24.5366 | 6.35019 | 0.027421 | up |
| TAF1D | 15.5802 | 3.00413 | 0.0186 | up |
| TAF1 | 6.65788 | 3.15199 | 0.024156 | up |
| SYNJ1 | 8.69795 | 3.38445 | 0.044852 | up |
| SYDE2 | 3.48291 | 1.0834 | 0.009746 | up |
| SUGT1 | 36.7608 | 10.6397 | 0.004024 | up |
| SSBP2 | 49.5963 | 24.6347 | 0.004024 | up |
| SRSF8 | 6.75844 | 2.44183 | 0.044852 | up |
| SRPRB | 16.0296 | 7.21137 | 0.039327 | up |
| SRP54 | 7.24989 | 3.04985 | 0.026025 | up |
| SPTY2D1 | 8.86844 | 2.99632 | 0.004024 | up |
| SPOPL | 6.07677 | 1.76272 | 0.004024 | up |
| SP8 | 6.87131 | 2.71016 | 0.004024 | up |
| SOCS6 | 6.72868 | 1.92785 | 0.004024 | up |
| SNX18 | 9.59003 | 3.56861 | 0.004024 | up |
| SNX1 | 18.0484 | 6.63474 | 0.035058 | up |
| SNHG14 | 9.63083 | 2.63652 | 0.016692 | up |
| SND1 | 70.9358 | 31.7291 | 0.004024 | up |
| SMOX | 6.74535 | 3.13031 | 0.037864 | up |
| SMAD7 | 3.20742 | 1.10856 | 0.007147 | up |
| SLC7A5 | 92.0583 | 13.6827 | 0.004024 | up |
| SLC7A11 | 10.9584 | 2.16692 | 0.004024 | up |
| SLC3A2 | 154.659 | 29.9131 | 0.004024 | up |
| SLC30A1 | 15.9338 | 5.75521 | 0.004024 | up |
| SLC25A25 | 12.2661 | 4.94873 | 0.004024 | up |
| SLC1A4 | 20.4886 | 6.5869 | 0.004024 | up |
| SLC17A5 | 3.92726 | 1.82053 | 0.027421 | up |
| SKIL | 18.5794 | 6.66508 | 0.007147 | up |
| SHMT2 | 40.5577 | 11.8049 | 0.004024 | up |
| SHISA2 | 4.91367 | 1.97914 | 0.004024 | up |
| SGTB | 11.4609 | 5.69139 | 0.027421 | up |
| SGPP1 | 7.40099 | 3.07002 | 0.0186 | up |
| SETBP1 | 6.71435 | 3.23081 | 0.020597 | up |
| SESN2 | 35.7077 | 9.35309 | 0.004024 | up |
| SERTAD1 | 5.65397 | 1.78211 | 0.004024 | up |
| SEC24D | 12.7296 | 1.11063 | 0.004024 | up |
| SEC23IP | 3.71526 | 0.422385 | 0.007147 | up |
| SEC23B | 25.6865 | 8.69026 | 0.009746 | up |
| SEC23A | 12.6328 | 4.41554 | 0.037864 | up |
| SCYL2 | 20.579 | 9.33192 | 0.046221 | up |
| SCAI | 7.91255 | 3.74648 | 0.004024 | up |
| SBNO1 | 7.77496 | 2.04156 | 0.029065 | up |
| SBNO1 | 7.42346 | 3.12694 | 0.004024 | up |
| SARS | 160.949 | 68.4641 | 0.004024 | up |
| RSBN1L | 7.91617 | 2.24913 | 0.033564 | up |
| RRAS2 | 17.5076 | 7.93581 | 0.016692 | up |
| RP5-864K19.7 | 13.5006 | 5.8118 | 0.027421 | up |
| RP4-665J23.1 | 21.7002 | 9.37555 | 0.004024 | up |
| RP11-66D17.5 | 2.06595 | 0 | 0.037864 | up |
| RP11-649E7.5 | 23.5426 | 11.4248 | 0.004024 | up |
| RP11-48B3.3 | 1.87692 | 0.694207 | 0.004024 | up |
| RP11-442H21.2 | 97.0084 | 22.8625 | 0.004024 | up |
| RP11-42F12.1 | 3.26543 | 1.22034 | 0.004024 | up |
| RP11-379K17.12 | 26.1788 | 8.95013 | 0.009746 | up |
| RP11-23N2.4 | 8.45101 | 3.03509 | 0.004024 | up |
| RP11-196G18.22 | 8.69553 | 3.50362 | 0.007147 | up |
| RNF41 | 11.1715 | 3.88329 | 0.007147 | up |
| RNF19B | 8.02003 | 2.57838 | 0.016692 | up |
| RNF169 | 3.87324 | 0.765764 | 0.020597 | up |
| RNF152 | 9.16745 | 3.20492 | 0.004024 | up |
| RNF138 | 29.6278 | 11.1961 | 0.004024 | up |
| RND3 | 45.155 | 21.9727 | 0.004024 | up |
| RND1 | 5.13211 | 1.68143 | 0.004024 | up |
| RN7SK | 36.7478 | 8.92139 | 0.016692 | up |
| RLIM | 26.1652 | 13.0169 | 0.004024 | up |
| RIOK3 | 16.9977 | 5.0285 | 0.04218 | up |
| RHEB | 2.87512 | 1.1564 | 0.035058 | up |
| RGS3 | 13.0349 | 6.25688 | 0.012156 | up |
| REPS1 | 13.8096 | 6.81154 | 0.043573 | up |
| RBM5 | 35.4866 | 14.616 | 0.0186 | up |
| RBM39 | 47.9036 | 12.2031 | 0.004024 | up |
| RBM25 | 36.3257 | 14.7246 | 0.004024 | up |
| RBM15 | 12.4041 | 5.12629 | 0.004024 | up |
| RAPGEF2 | 9.59808 | 3.07082 | 0.039327 | up |
| RABGAP1 | 9.55497 | 3.17186 | 0.020597 | up |
| RAB5A | 47.7934 | 22.0938 | 0.004024 | up |
| RAB39B | 10.0509 | 4.79412 | 0.004024 | up |
| PTPN1 | 11.0177 | 3.12384 | 0.004024 | up |
| PTP4A1 | 163.845 | 62.213 | 0.004024 | up |
| PSPH | 11.2879 | 1.83049 | 0.004024 | up |
| PSAT1 | 131.862 | 54.294 | 0.004024 | up |
| PRR14L | 4.94006 | 2.12163 | 0.033564 | up |
| PRPF38B | 28.7137 | 13.1211 | 0.004024 | up |
| PRNP | 9.14384 | 4.10105 | 0.027421 | up |
| PRKACB | 8.35154 | 1.69669 | 0.033564 | up |
| PRKAA2 | 3.62615 | 1.12499 | 0.007147 | up |
| PPP4R1L | 2.19956 | 0.964861 | 0.044852 | up |
| PPP2R5E | 15.3882 | 7.0602 | 0.044852 | up |
| PPP1R15B | 46.2407 | 17.741 | 0.004024 | up |
| PPP1CB | 61.5109 | 28.2272 | 0.004024 | up |
| PPIL4 | 34.7476 | 12.0687 | 0.004024 | up |
| PPIG | 10.5442 | 2.82961 | 0.012156 | up |
| POLR2A | 98.7814 | 34.3573 | 0.004024 | up |
| PMAIP1 | 24.8396 | 7.53253 | 0.004024 | up |
| PLK2 | 39.05 | 18.3767 | 0.004024 | up |
| PLEKHG5 | 2.59861 | 0.929622 | 0.047519 | up |
| PLEKHF2 | 10.9716 | 5.04249 | 0.004024 | up |
| PLEKHB2 | 17.7567 | 8.49436 | 0.020597 | up |
| PLCH1 | 13.9042 | 2.46927 | 0.004024 | up |
| PLCD1 | 9.15568 | 4.29004 | 0.004024 | up |
| PLAG1 | 4.52759 | 1.34304 | 0.016692 | up |
| PIM3 | 19.8758 | 9.91433 | 0.004024 | up |
| PIM2 | 2.66476 | 0.694391 | 0.0186 | up |
| PIKFYVE | 5.54859 | 1.59847 | 0.037864 | up |
| PHGDH | 166.915 | 65.3942 | 0.004024 | up |
| PHC3 | 13.0822 | 4.42795 | 0.004024 | up |
| PER1 | 11.3346 | 4.69936 | 0.047519 | up |
| PEG3 | 65.3913 | 26.795 | 0.039327 | up |
| PDP2 | 2.95756 | 1.39718 | 0.022555 | up |
| PCLO | 3.37104 | 1.03147 | 0.040714 | up |
| PCDH20 | 1.81208 | 0.32026 | 0.007147 | up |
| PCDH19 | 7.43184 | 2.68485 | 0.004024 | up |
| PAPOLG | 6.4465 | 2.38754 | 0.014616 | up |
| PAN3 | 9.49347 | 3.87451 | 0.009746 | up |
| PAN3 | 3.49032 | 1.5653 | 0.048958 | up |
| OTUD1 | 11.3213 | 3.13873 | 0.004024 | up |
| OSGIN2 | 5.8514 | 2.65656 | 0.004024 | up |
| OSER1 | 15.8972 | 6.73751 | 0.0186 | up |
| OSBP | 14.2732 | 6.25167 | 0.004024 | up |
| NUDT4 | 28.3161 | 11.1271 | 0.007147 | up |
| NUDT4 | 25.3474 | 10.466 | 0.004024 | up |
| NUCB2 | 19.117 | 6.15095 | 0.004024 | up |
| NRIP1 | 6.3354 | 1.8796 | 0.004024 | up |
| NR4A3 | 2.88913 | 0.40965 | 0.009746 | up |
| NOG | 3.06976 | 0.69458 | 0.046221 | up |
| NMD3 | 19.7047 | 9.38712 | 0.004024 | up |
| NKTR | 17.9824 | 7.77679 | 0.009746 | up |
| NFIL3 | 13.1109 | 6.42884 | 0.004024 | up |
| NEU1 | 24.2693 | 10.6971 | 0.009746 | up |
| NCK1 | 13.3615 | 3.83574 | 0.004024 | up |
| NANS | 11.1176 | 3.89954 | 0.004024 | up |
| NAMPT | 12.6786 | 5.90459 | 0.004024 | up |
| NADK2-AS1 | 5.08096 | 1.84208 | 0.004024 | up |
| NADK2 | 13.7188 | 4.17158 | 0.004024 | up |
| MYO9B | 4.94879 | 2.37025 | 0.0186 | up |
| MVK | 14.8055 | 6.84383 | 0.039327 | up |
| MMS19 | 17.3516 | 8.45999 | 0.012156 | up |
| MBNL2 | 5.54971 | 1.87864 | 0.004024 | up |
| MARS | 63.6958 | 27.2678 | 0.004024 | up |
| MAP7D1 | 40.826 | 19.6006 | 0.004024 | up |
| MAGEL2 | 14.7393 | 5.20796 | 0.004024 | up |
| MAFB | 3.9319 | 1.58959 | 0.004024 | up |
| LMO4 | 70.5698 | 27.3886 | 0.004024 | up |
| LINC00888 | 19.572 | 5.53516 | 0.004024 | up |
| LIN7C | 19.2714 | 7.2447 | 0.004024 | up |
| KMT2C | 8.4852 | 1.4452 | 0.040714 | up |
| KMT2C | 5.31752 | 1.85637 | 0.007147 | up |
| KLHL29 | 7.7026 | 2.45358 | 0.027421 | up |
| KLHL28 | 16.5131 | 7.48678 | 0.004024 | up |
| KLHL11 | 10.123 | 3.7128 | 0.004024 | up |
| KLHDC8B | 34.8856 | 17.143 | 0.004024 | up |
| KLF15 | 10.5999 | 1.66818 | 0.004024 | up |
| KLF10 | 15.6402 | 4.38326 | 0.004024 | up |
| KIDINS220 | 27.9353 | 10.7179 | 0.016692 | up |
| KIAA1324L | 6.14501 | 2.59443 | 0.043573 | up |
| KDM5C | 7.88384 | 2.36966 | 0.022555 | up |
| KCNT2 | 5.00351 | 2.23151 | 0.007147 | up |
| KCNJ4 | 7.64132 | 3.54946 | 0.030479 | up |
| JDP2 | 8.55961 | 1.86776 | 0.004024 | up |
| ISCU | 50.353 | 24.4687 | 0.004024 | up |
| IRS2 | 13.1161 | 5.26979 | 0.004024 | up |
| INSIG1 | 74.7067 | 34.3649 | 0.004024 | up |
| INPP5B | 5.13669 | 1.24589 | 0.009746 | up |
| INO80D | 9.13036 | 3.34451 | 0.004024 | up |
| INHBE | 2.74233 | 0.40021 | 0.047519 | up |
| ING2 | 11.8664 | 5.04236 | 0.007147 | up |
| IDI1 | 48.0704 | 18.5201 | 0.032149 | up |
| IARS | 13.9563 | 4.14473 | 0.004024 | up |
| IARS | 50.0008 | 21.2833 | 0.004024 | up |
| HYOU1 | 55.0236 | 5.28065 | 0.004024 | up |
| HSPA9 | 88.251 | 43.2571 | 0.004024 | up |
| HMGCR | 51.7209 | 24.0657 | 0.029065 | up |
| HM13 | 88.5955 | 37.63 | 0.004024 | up |
| HIST1H2BE | 3.88057 | 1.25801 | 0.004024 | up |
| HIST1H2AC | 7.09805 | 2.63761 | 0.009746 | up |
| HIBADH | 10.305 | 3.95098 | 0.040714 | up |
| HEY1 | 4.43344 | 1.67517 | 0.016692 | up |
| HERPUD1 | 15.9474 | 1.57483 | 0.035058 | up |
| HERPUD1 | 48.4607 | 4.9494 | 0.004024 | up |
| HELZ | 11.3836 | 4.73012 | 0.04218 | up |
| HDAC1 | 9.77896 | 3.98804 | 0.036539 | up |
| GUK1 | 7.34697 | 1.89762 | 0.009746 | up |
| GPCPD1 | 14.8062 | 5.55464 | 0.009746 | up |
| GOLGA5 | 18.4596 | 7.87084 | 0.004024 | up |
| GOLGA4 | 10.252 | 3.95224 | 0.004024 | up |
| GFPT1 | 24.8381 | 11.2534 | 0.004024 | up |
| GDF15 | 11.3394 | 2.43496 | 0.004024 | up |
| GDAP1 | 13.2139 | 4.35162 | 0.022555 | up |
| GARS | 93.0078 | 36.2854 | 0.004024 | up |
| GADD45B | 9.92209 | 2.96863 | 0.004024 | up |
| FRY | 3.70458 | 1.45894 | 0.004024 | up |
| FRS2 | 13.4106 | 4.89932 | 0.036539 | up |
| FOXJ3 | 9.92603 | 4.19457 | 0.020597 | up |
| FNIP1 | 6.61493 | 1.43868 | 0.030479 | up |
| FGFR1OP2 | 17.0345 | 4.80323 | 0.012156 | up |
| FBRS | 11.9455 | 2.48738 | 0.004024 | up |
| FAR1 | 8.4657 | 3.33323 | 0.014616 | up |
| FAM167A | 5.4317 | 1.5688 | 0.035058 | up |
| EXOC8 | 6.65273 | 2.3554 | 0.009746 | up |
| EVI5L | 7.52769 | 3.07643 | 0.037864 | up |
| ERO1B | 2.10605 | 0.328472 | 0.004024 | up |
| EPS15 | 21.1211 | 9.07451 | 0.004024 | up |
| EPHX1 | 7.72053 | 2.71488 | 0.007147 | up |
| EMC2 | 14.1434 | 6.49747 | 0.047519 | up |
| EIF4EBP1 | 37.6618 | 9.69957 | 0.004024 | up |
| EIF1 | 369.511 | 156.862 | 0.004024 | up |
| EAF1 | 6.24304 | 2.19709 | 0.046221 | up |
| DYRK2 | 12.7395 | 4.65707 | 0.037864 | up |
| DUSP14 | 18.4079 | 6.48046 | 0.036539 | up |
| DPF2 | 15.2646 | 6.30359 | 0.043573 | up |
| DNAJC3 | 14.6847 | 4.56135 | 0.004024 | up |
| DNAJC1 | 14.3502 | 7.1652 | 0.004024 | up |
| DNAJB9 | 67.6108 | 19.3938 | 0.004024 | up |
| DNAJB11 | 10.9276 | 3.88971 | 0.007147 | up |
| DNAJA3 | 15.3571 | 6.71557 | 0.004024 | up |
| DIS3 | 8.61469 | 3.00069 | 0.014616 | up |
| DIRAS2 | 4.63049 | 1.68397 | 0.004024 | up |
| DGKD | 5.78011 | 1.44143 | 0.004024 | up |
| DDX3Y | 14.8177 | 2.72831 | 0.004024 | up |
| DDIT4 | 28.4136 | 6.39764 | 0.004024 | up |
| DAPK3 | 11.3772 | 3.95858 | 0.040714 | up |
| CTH | 3.46297 | 0.790295 | 0.004024 | up |
| CTH | 11.2451 | 2.68376 | 0.009746 | up |
| CTC-444N24.8 | 5.21357 | 2.07338 | 0.027421 | up |
| CTC-444N24.7 | 6.56108 | 2.83168 | 0.004024 | up |
| CSRNP1 | 7.09156 | 2.72054 | 0.004024 | up |
| CSNK2A1 | 35.9296 | 16.8856 | 0.024156 | up |
| CREBRF | 10.4839 | 4.48548 | 0.004024 | up |
| CREB3L2 | 13.4053 | 2.60347 | 0.004024 | up |
| COG3 | 17.1623 | 7.38022 | 0.004024 | up |
| CLK1 | 31.9702 | 8.30936 | 0.004024 | up |
| CLASRP | 17.8385 | 5.20728 | 0.014616 | up |
| CHORDC1 | 7.81269 | 3.68576 | 0.036539 | up |
| CHD1 | 6.18085 | 1.49641 | 0.022555 | up |
| CHAC1 | 21.9038 | 1.18592 | 0.030479 | up |
| CEBPB | 21.6349 | 2.48016 | 0.004024 | up |
| CDC42EP1 | 3.51627 | 0.667904 | 0.004024 | up |
| CCSER1 | 3.22817 | 1.33218 | 0.039327 | up |
| CCNT2 | 22.6849 | 10.7178 | 0.009746 | up |
| CCDC174 | 9.44576 | 3.81297 | 0.004024 | up |
| CBX4 | 34.9579 | 13.5365 | 0.004024 | up |
| CARS | 10.186 | 2.12427 | 0.004024 | up |
| CARS | 34.822 | 9.94765 | 0.004024 | up |
| CAMSAP1 | 13.0782 | 5.47986 | 0.032149 | up |
| CAMKMT | 7.68583 | 3.79147 | 0.029065 | up |
| CA2 | 42.7651 | 18.4952 | 0.004024 | up |
| C18orf25 | 10.055 | 4.56833 | 0.0186 | up |
| C16orf87 | 22.3985 | 9.00547 | 0.007147 | up |
| C16orf72 | 14.7979 | 5.47276 | 0.007147 | up |
| BTBD10 | 17.7128 | 6.48499 | 0.004024 | up |
| BMP2 | 1.6975 | 0.70988 | 0.037864 | up |
| BHLHE40 | 4.72113 | 0.871519 | 0.004024 | up |
| BCOR | 9.48968 | 3.54474 | 0.004024 | up |
| BCL9L | 7.62209 | 2.86068 | 0.004024 | up |
| BCL6 | 2.05989 | 0.841914 | 0.007147 | up |
| BBS12 | 3.21386 | 1.10407 | 0.033564 | up |
| BBC3 | 24.9293 | 7.74762 | 0.004024 | up |
| BBC3 | 13.5394 | 5.40709 | 0.04218 | up |
| BAIAP2 | 26.7338 | 12.204 | 0.004024 | up |
| ATXN2L | 28.2632 | 11.4921 | 0.004024 | up |
| ATP6V1B2 | 10.3259 | 2.55984 | 0.024156 | up |
| ATG2B | 3.1196 | 0.547253 | 0.014616 | up |
| ATG2A | 3.78251 | 1.52017 | 0.0186 | up |
| ATF3 | 28.2811 | 11.0207 | 0.004024 | up |
| ASNS | 143.897 | 35.4081 | 0.004024 | up |
| ASCC2 | 8.92547 | 4.15875 | 0.04218 | up |
| ARID4A | 5.83135 | 1.1384 | 0.035058 | up |
| ARHGEF10L | 4.08456 | 0.949536 | 0.032149 | up |
| ARHGAP23 | 6.54782 | 3.07129 | 0.035058 | up |
| ARFGAP3 | 37.2501 | 18.5913 | 0.004024 | up |
| AQR | 9.02206 | 2.36475 | 0.024156 | up |
| ANKRD33B | 3.30003 | 0.878766 | 0.004024 | up |
| ALKBH1 | 5.89389 | 1.93427 | 0.0186 | up |
| ALAS1 | 9.94489 | 3.50178 | 0.035058 | up |
| AKT3 | 28.4524 | 13.3746 | 0.036539 | up |
| AKAP8L | 21.5841 | 7.47332 | 0.047519 | up |
| AKAP17A | 10.7053 | 4.15053 | 0.007147 | up |
| AKAP17A | 17.3773 | 7.27257 | 0.004024 | up |
| AKAP10 | 9.88228 | 4.66623 | 0.004024 | up |
| AGTPBP1 | 15.8626 | 7.85407 | 0.024156 | up |
| AEBP2 | 10.8315 | 3.31998 | 0.012156 | up |
| ADM2 | 2.27542 | 0.265531 | 0.004024 | up |
| ADCY9 | 4.13695 | 1.97236 | 0.0186 | up |
| ACSL3 | 50.4711 | 15.6379 | 0.004024 | up |
| ACBD3 | 19.7588 | 7.83042 | 0.004024 | up |
| AC144652.1 | 4.19838 | 1.7021 | 0.040714 | up |
| ABCA1 | 11.3319 | 4.43075 | 0.033564 | up |
| AARS | 57.2526 | 24.0056 | 0.004024 | up |
| ZNF730 | 1.16022 | 4.34875 | 0.004024 | down |
| ZNF514 | 1.85325 | 6.1033 | 0.016692 | down |
| ZNF271P | 2.86743 | 5.76371 | 0.007147 | down |
| ZDHHC7 | 5.09039 | 10.8499 | 0.009746 | down |
| YLPM1 | 5.68985 | 13.164 | 0.009746 | down |
| YEATS4 | 9.10395 | 18.7056 | 0.004024 | down |
| XRCC2 | 0.561555 | 3.4567 | 0.020597 | down |
| WSB1 | 346.82 | 1242.38 | 0.004024 | down |
| WNT8B | 7.56812 | 27.9691 | 0.004024 | down |
| WNT7B | 30.8065 | 77.1324 | 0.004024 | down |
| VPS13C | 1.27968 | 4.18587 | 0.026025 | down |
| VCAN | 2.89099 | 10.0174 | 0.004024 | down |
| UTRN | 2.16239 | 4.64591 | 0.030479 | down |
| URM1 | 4.30725 | 8.98584 | 0.004024 | down |
| UQCRHL | 1.34922 | 3.63692 | 0.012156 | down |
| UNG | 2.70544 | 11.8503 | 0.004024 | down |
| UBE2G2 | 6.79708 | 19.5093 | 0.039327 | down |
| UBE2C | 28.4137 | 65.7732 | 0.004024 | down |
| U2SURP | 2.92606 | 17.7177 | 0.004024 | down |
| TYMS | 22.3667 | 53.727 | 0.004024 | down |
| TUG1 | 5.5138 | 17.1435 | 0.014616 | down |
| TUBB6 | 6.58767 | 16.1811 | 0.022555 | down |
| TUBB4B | 64.7486 | 143.185 | 0.004024 | down |
| TUBA1B | 144.73 | 315.616 | 0.0186 | down |
| TTF2 | 0.462615 | 1.96079 | 0.004024 | down |
| TSPAN14 | 5.88605 | 12.162 | 0.016692 | down |
| TRPM7 | 0.6641 | 2.42268 | 0.043573 | down |
| TRIOBP | 3.17556 | 7.77989 | 0.030479 | down |
| TRIOBP | 2.02972 | 4.67683 | 0.027421 | down |
| TRIM45 | 4.22414 | 14.6015 | 0.004024 | down |
| TRIL | 2.43237 | 5.09793 | 0.004024 | down |
| TPM1 | 6.78823 | 22.7461 | 0.032149 | down |
| TPBG | 2.50254 | 36.8761 | 0.004024 | down |
| TPBG | 2.78141 | 14.0638 | 0.022555 | down |
| TPBG | 34.8776 | 118.148 | 0.004024 | down |
| TOP2A | 28.1619 | 68.9765 | 0.004024 | down |
| TNFRSF19 | 1.57282 | 5.69621 | 0.004024 | down |
| TMUB2 | 3.63684 | 7.86184 | 0.035058 | down |
| TMPO | 15.1125 | 36.2344 | 0.004024 | down |
| TMEM67 | 4.33745 | 9.02359 | 0.044852 | down |
| TMEM254 | 7.12288 | 16.5217 | 0.014616 | down |
| TMEM2 | 11.8605 | 26.8321 | 0.004024 | down |
| TMEM168 | 0.567204 | 3.10338 | 0.009746 | down |
| TK1 | 4.03405 | 8.87372 | 0.0186 | down |
| TIMELESS | 2.26031 | 5.53699 | 0.043573 | down |
| THBS1 | 18.1658 | 37.3234 | 0.007147 | down |
| TCTN2 | 4.65789 | 9.75073 | 0.007147 | down |
| TCF19 | 1.33557 | 4.04472 | 0.016692 | down |
| TAS2R5 | 0.561001 | 1.84787 | 0.007147 | down |
| TAF8 | 1.26127 | 2.69335 | 0.035058 | down |
| SRSF5 | 21.1184 | 57.1218 | 0.004024 | down |
| SRSF5 | 50.0059 | 130.723 | 0.004024 | down |
| SPAG9 | 9.30006 | 24.5531 | 0.030479 | down |
| SOX1 | 5.69118 | 12.3629 | 0.009746 | down |
| SOCS3 | 3.8835 | 13.5767 | 0.004024 | down |
| SNRPGP10 | 2.62857 | 5.34304 | 0.024156 | down |
| SNRPA | 15.4261 | 49.6874 | 0.004024 | down |
| SMC2 | 4.26006 | 9.71432 | 0.004024 | down |
| SLC44A5 | 1.06574 | 4.28101 | 0.04218 | down |
| SKP2 | 7.29607 | 15.4008 | 0.004024 | down |
| SKA3 | 0.871568 | 3.9826 | 0.04218 | down |
| SGOL2 | 1.73502 | 5.26475 | 0.007147 | down |
| SGOL2 | 1.26459 | 3.51045 | 0.004024 | down |
| SFRP2 | 128.873 | 387.368 | 0.004024 | down |
| SFPQ | 28.5248 | 119.433 | 0.004024 | down |
| SEPT8 | 4.89934 | 12.5319 | 0.007147 | down |
| SEPT10 | 1.28167 | 6.35826 | 0.014616 | down |
| SEMA6A | 16.7823 | 41.7668 | 0.012156 | down |
| SDC1 | 3.42435 | 10.6435 | 0.004024 | down |
| SCUBE1 | 4.43183 | 14.1879 | 0.004024 | down |
| SCARA3 | 1.01622 | 2.4867 | 0.004024 | down |
| SAPCD2 | 2.45051 | 7.12607 | 0.004024 | down |
| SALL2 | 12.2183 | 32.9858 | 0.004024 | down |
| RSPO2 | 4.35245 | 10.9199 | 0.009746 | down |
| RRM2 | 4.36454 | 30.0612 | 0.004024 | down |
| RRM1 | 18.1079 | 36.8448 | 0.004024 | down |
| RPS27P25 | 1.99993 | 4.43959 | 0.007147 | down |
| RPS27L | 23.0146 | 83.5052 | 0.004024 | down |
| RP6-24A23.7 | 2.39432 | 9.81096 | 0.004024 | down |
| RP6-24A23.6 | 1.55527 | 7.57316 | 0.004024 | down |
| RP5-1074L1.4 | 2.14648 | 9.22546 | 0.026025 | down |
| RP3-395M20.12 | 4.56931 | 13.6344 | 0.004024 | down |
| RP1-39G22.7 | 4.5345 | 10.5115 | 0.007147 | down |
| RP11-725P16.2 | 3.2834 | 9.33207 | 0.004024 | down |
| RP11-65F13.3 | 0.797863 | 2.51771 | 0.009746 | down |
| RP11-567M16.6 | 1.69381 | 4.08085 | 0.033564 | down |
| RP11-566E18.1 | 4.00295 | 8.18627 | 0.004024 | down |
| RP11-390E23.6 | 1.26704 | 3.75118 | 0.024156 | down |
| RP11-381E24.1 | 1.24387 | 4.3813 | 0.004024 | down |
| RP11-307E17.8 | 1.66673 | 3.47127 | 0.044852 | down |
| RP11-212P7.2 | 1.55108 | 3.10385 | 0.004024 | down |
| RP11-191L17.1 | 0 | 1.8122 | 0.040714 | down |
| RP11-119K6.6 | 3.7994 | 11.5596 | 0.022555 | down |
| RP11-1094M14.11 | 3.31754 | 8.13287 | 0.0186 | down |
| RP11-1055B8.4 | 3.92713 | 11.7277 | 0.004024 | down |
| RP11-1055B8.4 | 4.87795 | 11.753 | 0.004024 | down |
| RMI2 | 5.3385 | 13.5581 | 0.004024 | down |
| RING1 | 13.8994 | 31.3941 | 0.004024 | down |
| RFC3 | 3.63157 | 10.1144 | 0.004024 | down |
| RBMX | 25.834 | 63.6528 | 0.030479 | down |
| RBM14 | 10.9792 | 29.6931 | 0.004024 | down |
| RBBP7 | 10.2617 | 28.1899 | 0.004024 | down |
| RALGDS | 4.53212 | 13.2603 | 0.035058 | down |
| RAD51-AS1 | 0.899773 | 2.53199 | 0.004024 | down |
| RAD51AP1 | 1.91322 | 6.74123 | 0.004024 | down |
| QRSL1 | 2.34546 | 5.09853 | 0.016692 | down |
| PTN | 111.661 | 242.633 | 0.004024 | down |
| PTGIS | 0.562045 | 2.84154 | 0.007147 | down |
| PSPC1 | 6.43551 | 24.6482 | 0.004024 | down |
| PSMA3-AS1 | 9.69453 | 31.8737 | 0.026025 | down |
| PRPS2 | 2.83326 | 7.40118 | 0.004024 | down |
| PRMT8 | 1.29138 | 5.11119 | 0.004024 | down |
| PRKDC | 4.52439 | 11.2427 | 0.012156 | down |
| PPWD1 | 3.48512 | 10.2648 | 0.009746 | down |
| PPIF | 5.26635 | 12.4137 | 0.004024 | down |
| PODXL | 6.669 | 19.1968 | 0.004024 | down |
| PLP1 | 2.14138 | 5.57544 | 0.0186 | down |
| PLK1 | 13.4551 | 28.6335 | 0.004024 | down |
| PLAGL1 | 6.41895 | 24.132 | 0.004024 | down |
| PKI55 | 5.85646 | 12.0986 | 0.016692 | down |
| PIANP | 5.47445 | 12.0047 | 0.016692 | down |
| PHF6 | 6.18876 | 13.565 | 0.020597 | down |
| PFN2 | 34.6327 | 112.14 | 0.004024 | down |
| PDLIM1 | 1.92085 | 4.46117 | 0.020597 | down |
| PDDC1 | 4.6784 | 12.6929 | 0.004024 | down |
| PCNA | 48.3358 | 113.982 | 0.004024 | down |
| PBXIP1 | 4.06532 | 12.1396 | 0.004024 | down |
| PARP4 | 4.02643 | 8.2312 | 0.004024 | down |
| PABPN1 | 8.45469 | 70.4926 | 0.004024 | down |
| OSBPL7 | 1.34676 | 3.1428 | 0.024156 | down |
| ORC1 | 2.22599 | 5.13813 | 0.004024 | down |
| OLIG3 | 2.44017 | 5.81251 | 0.004024 | down |
| OGT | 5.28394 | 22.5152 | 0.007147 | down |
| OBSL1 | 2.05331 | 8.6787 | 0.004024 | down |
| NUSAP1 | 14.8099 | 37.0109 | 0.016692 | down |
| NUP62CL | 0.685053 | 1.74861 | 0.004024 | down |
| NUP107 | 6.11102 | 13.1244 | 0.027421 | down |
| NSMCE4A | 5.43674 | 12.2821 | 0.033564 | down |
| NR2C1 | 0.896495 | 6.63052 | 0.033564 | down |
| NME4 | 50.1932 | 102.778 | 0.004024 | down |
| NFATC2IP | 2.05968 | 6.0388 | 0.027421 | down |
| NES | 39.0795 | 87.1707 | 0.004024 | down |
| NEMP1 | 0.671862 | 4.45865 | 0.032149 | down |
| NCAPD2 | 7.32168 | 18.0732 | 0.004024 | down |
| NAT14 | 15.5502 | 31.3243 | 0.004024 | down |
| NASP | 11.3014 | 38.0697 | 0.004024 | down |
| MZF1 | 7.08716 | 16.1825 | 0.020597 | down |
| MURC | 3.90275 | 7.92573 | 0.007147 | down |
| MOGS | 5.00643 | 12.1796 | 0.016692 | down |
| MMS22L | 0.774481 | 3.1879 | 0.014616 | down |
| MMRN1 | 4.71513 | 18.8114 | 0.004024 | down |
| MMP2 | 1.13353 | 3.04897 | 0.026025 | down |
| MKRN3 | 4.57228 | 12.2722 | 0.004024 | down |
| MKRN3 | 1.53357 | 3.96661 | 0.007147 | down |
| MIS18BP1 | 2.38627 | 6.3926 | 0.004024 | down |
| MFNG | 2.77077 | 7.72116 | 0.004024 | down |
| MFGE8 | 5.14739 | 25.9216 | 0.004024 | down |
| MEGF6 | 1.94491 | 5.31956 | 0.009746 | down |
| MEGF6 | 1.37238 | 2.90464 | 0.046221 | down |
| MDC1 | 4.52623 | 9.72945 | 0.004024 | down |
| MCM6 | 7.01877 | 25.6708 | 0.004024 | down |
| MCM5 | 1.41192 | 8.72169 | 0.004024 | down |
| MCM5 | 0.69359 | 3.09377 | 0.04218 | down |
| MCM5 | 5.02933 | 11.1911 | 0.024156 | down |
| MCM4 | 10.0887 | 35.0801 | 0.004024 | down |
| MCM3 | 8.23956 | 25.0627 | 0.004024 | down |
| MCM2 | 7.43658 | 22.7438 | 0.004024 | down |
| MAT2A | 17.0194 | 64.733 | 0.004024 | down |
| MAPK15 | 0.635832 | 4.25818 | 0.004024 | down |
| MAPK15 | 0.912262 | 4.68571 | 0.026025 | down |
| MAPK15 | 1.13102 | 5.78746 | 0.014616 | down |
| MAPK11 | 7.11065 | 20.3135 | 0.004024 | down |
| MAD2L1 | 2.07949 | 5.21348 | 0.004024 | down |
| LUC7L3 | 2.7293 | 10.5475 | 0.009746 | down |
| LUC7L3 | 18.533 | 47.3282 | 0.004024 | down |
| LRRC37A4P | 1.71079 | 7.46151 | 0.009746 | down |
| LPAR4 | 2.73105 | 6.54061 | 0.016692 | down |
| LOXL2 | 4.04956 | 12.4583 | 0.024156 | down |
| LOXL1 | 2.57912 | 7.44762 | 0.009746 | down |
| LINC01521 | 1.42186 | 3.95207 | 0.004024 | down |
| LINC01021 | 2.05879 | 8.42402 | 0.004024 | down |
| LINC01021 | 4.26332 | 10.4791 | 0.004024 | down |
| LINC00599 | 2.03121 | 12.2379 | 0.014616 | down |
| LINC00342 | 4.96873 | 10.2194 | 0.004024 | down |
| LHX5 | 14.9452 | 39.0171 | 0.004024 | down |
| LHX5 | 11.6215 | 25.3097 | 0.012156 | down |
| LENG8 | 20.6379 | 60.0585 | 0.004024 | down |
| LEMD1 | 1.96915 | 5.7999 | 0.004024 | down |
| KLHDC8A | 14.2328 | 39.7972 | 0.004024 | down |
| KLF4 | 2.66764 | 7.84418 | 0.0186 | down |
| KIFC1 | 7.15256 | 20.8782 | 0.004024 | down |
| KIFC1 | 3.4761 | 8.38789 | 0.007147 | down |
| KIF22 | 2.59166 | 6.84858 | 0.046221 | down |
| KIF15 | 3.94801 | 10.2489 | 0.012156 | down |
| KIF11 | 9.58071 | 19.612 | 0.004024 | down |
| KIAA1456 | 0.471472 | 2.12357 | 0.004024 | down |
| KDELC2 | 1.7075 | 5.5197 | 0.004024 | down |
| KAT2A | 6.54388 | 15.3259 | 0.016692 | down |
| JRK | 0.758756 | 4.46995 | 0.004024 | down |
| JAM2 | 5.29165 | 12.317 | 0.027421 | down |
| IPO5P1 | 2.36399 | 12.8307 | 0.004024 | down |
| INF2 | 1.04942 | 2.4761 | 0.020597 | down |
| INCENP | 1.41274 | 5.09441 | 0.029065 | down |
| ILF3-AS1 | 3.16481 | 8.5499 | 0.004024 | down |
| IL13RA1 | 1.5196 | 3.60367 | 0.007147 | down |
| IGFBP5 | 1.47803 | 3.30307 | 0.004024 | down |
| IGFBP4 | 4.56029 | 11.2069 | 0.004024 | down |
| IGF2 | 2.80013 | 7.68664 | 0.004024 | down |
| IFT81 | 1.45892 | 7.87966 | 0.032149 | down |
| IFT81 | 6.61778 | 16.2468 | 0.004024 | down |
| ID4 | 20.6234 | 64.937 | 0.004024 | down |
| HYAL2 | 11.9013 | 25.8385 | 0.004024 | down |
| HTRA1 | 5.50386 | 14.4048 | 0.033564 | down |
| HMGN3 | 22.7056 | 51.9705 | 0.004024 | down |
| HMGN2 | 210.548 | 453.864 | 0.004024 | down |
| HES6 | 34.0326 | 137.76 | 0.004024 | down |
| HES6 | 74.3054 | 167.205 | 0.004024 | down |
| HES4 | 22.368 | 47.0886 | 0.007147 | down |
| HEPH | 1.55221 | 5.80376 | 0.004024 | down |
| HELLS | 2.63925 | 8.07868 | 0.012156 | down |
| HCG18 | 1.68478 | 5.48467 | 0.004024 | down |
| H2AFX | 35.663 | 88.2856 | 0.004024 | down |
| GJA1 | 18.4857 | 40.6316 | 0.004024 | down |
| GINS3 | 0.892621 | 3.33414 | 0.030479 | down |
| GINS1 | 4.15267 | 10.1547 | 0.022555 | down |
| GEN1 | 3.0868 | 6.67699 | 0.029065 | down |
| GDPD2 | 1.03228 | 3.99929 | 0.032149 | down |
| GDF10 | 6.77815 | 26.0851 | 0.004024 | down |
| FZD3 | 8.27083 | 20.3958 | 0.004024 | down |
| FZD2 | 3.59101 | 13.2987 | 0.004024 | down |
| FOSB | 13.0874 | 39.2827 | 0.030479 | down |
| FOS | 242.504 | 532.271 | 0.004024 | down |
| FKBP10 | 12.7874 | 27.3152 | 0.029065 | down |
| FGFRL1 | 9.23711 | 23.9156 | 0.004024 | down |
| FGFR1OP | 8.77837 | 19.7003 | 0.004024 | down |
| FGFBP3 | 72.5107 | 160.917 | 0.004024 | down |
| FANCL | 3.3691 | 11.2625 | 0.0186 | down |
| FANCD2 | 1.69976 | 5.90051 | 0.004024 | down |
| FANCA | 2.22456 | 5.5447 | 0.027421 | down |
| FAM64A | 5.64337 | 12.944 | 0.024156 | down |
| FAM127C | 1.48405 | 3.07947 | 0.024156 | down |
| EXTL2 | 21.0593 | 46.6073 | 0.004024 | down |
| ESPL1 | 1.54164 | 3.18621 | 0.043573 | down |
| EPHB2 | 5.03669 | 14.59 | 0.0186 | down |
| ENGASE | 0.421933 | 4.11359 | 0.024156 | down |
| EMP2 | 2.62942 | 5.48779 | 0.004024 | down |
| EGR2 | 7.71325 | 15.9233 | 0.004024 | down |
| EGR1 | 242.069 | 576.893 | 0.004024 | down |
| E2F2 | 0.778744 | 3.06114 | 0.004024 | down |
| E2F1 | 6.20266 | 19.7272 | 0.004024 | down |
| DUT | 3.56195 | 12.668 | 0.036539 | down |
| DTL | 2.81775 | 8.53347 | 0.004024 | down |
| DSCC1 | 1.72177 | 4.8639 | 0.0186 | down |
| DPM3 | 10.5412 | 29.7953 | 0.035058 | down |
| DNPH1 | 7.8663 | 20.4799 | 0.004024 | down |
| DLL3 | 9.34783 | 30.2457 | 0.004024 | down |
| DLL3 | 23.4755 | 54.1957 | 0.004024 | down |
| DLGAP5 | 4.30793 | 13.5615 | 0.004024 | down |
| DLG3 | 3.93642 | 13.1853 | 0.0186 | down |
| DKFZP434I0714 | 1.12984 | 2.87013 | 0.026025 | down |
| DHFR | 7.93398 | 42.7593 | 0.004024 | down |
| DESI1 | 4.14859 | 8.43413 | 0.012156 | down |
| DEPDC1B | 0.861125 | 2.5699 | 0.030479 | down |
| DEPDC1B | 5.80585 | 15.0724 | 0.004024 | down |
| DDX11 | 3.96423 | 14.1097 | 0.004024 | down |
| DCAF16 | 4.44393 | 12.3 | 0.009746 | down |
| DARS2 | 1.96847 | 5.41129 | 0.020597 | down |
| CYP26B1 | 0.872966 | 2.84634 | 0.007147 | down |
| CTSF | 2.27033 | 9.2912 | 0.033564 | down |
| CTSC | 1.99888 | 6.93266 | 0.004024 | down |
| CTGF | 121.991 | 252.814 | 0.004024 | down |
| CTD-2228K2.7 | 3.04066 | 6.81203 | 0.0186 | down |
| CTC-246B18.8 | 2.58546 | 5.99579 | 0.037864 | down |
| CSRP2 | 29.9366 | 64.4518 | 0.004024 | down |
| CRISPLD1 | 9.78247 | 20.0318 | 0.004024 | down |
| CREG1 | 3.86463 | 8.08696 | 0.007147 | down |
| CRB2 | 3.1773 | 10.1932 | 0.033564 | down |
| CRB2 | 7.42678 | 15.6263 | 0.027421 | down |
| CPXM1 | 9.05972 | 25.3461 | 0.004024 | down |
| COL26A1 | 5.72099 | 13.0924 | 0.004024 | down |
| CLUH | 0.402079 | 2.91132 | 0.007147 | down |
| CKS1B | 38.5597 | 82.6686 | 0.004024 | down |
| CKAP2L | 1.3647 | 4.09944 | 0.007147 | down |
| CIT | 1.56408 | 3.21214 | 0.036539 | down |
| CHRAC1 | 2.47147 | 6.32411 | 0.048958 | down |
| CHD1L | 4.57029 | 13.2265 | 0.004024 | down |
| CHAF1A | 4.56395 | 11.1221 | 0.009746 | down |
| CH507-154B10.2 | 0.857137 | 3.8098 | 0.007147 | down |
| CENPM | 3.58118 | 10.7239 | 0.004024 | down |
| CENPK | 1.32324 | 3.80714 | 0.0186 | down |
| CENPF | 0.443803 | 3.5695 | 0.014616 | down |
| CENPF | 12.8119 | 27.4056 | 0.004024 | down |
| CENPC | 1.04872 | 3.63907 | 0.020597 | down |
| CDKN2C | 1.70758 | 4.48721 | 0.016692 | down |
| CDKL2 | 0.822694 | 2.13284 | 0.037864 | down |
| CDK1 | 15.839 | 37.6086 | 0.004024 | down |
| CDH24 | 3.30378 | 8.99606 | 0.004024 | down |
| CDH20 | 3.43852 | 15.0261 | 0.037864 | down |
| CDCA8 | 3.07631 | 11.9043 | 0.004024 | down |
| CDCA7 | 2.92068 | 6.88304 | 0.026025 | down |
| CDC45 | 2.60734 | 7.181 | 0.029065 | down |
| CDC20 | 18.3078 | 46.6792 | 0.004024 | down |
| CD248 | 0.586135 | 2.02057 | 0.009746 | down |
| CCNB2 | 19.9188 | 40.621 | 0.004024 | down |
| CCNB1 | 6.10951 | 25.7715 | 0.004024 | down |
| CCNB1 | 7.54004 | 25.0454 | 0.004024 | down |
| CCDC40 | 2.48454 | 6.00244 | 0.009746 | down |
| CASC5 | 1.22845 | 3.84573 | 0.004024 | down |
| CAMKV | 1.74653 | 6.57714 | 0.014616 | down |
| C4orf3 | 9.89012 | 22.8212 | 0.004024 | down |
| C2orf48 | 0.785953 | 2.46168 | 0.007147 | down |
| C2CD4C | 3.5066 | 8.45667 | 0.004024 | down |
| C1GALT1C1 | 3.55052 | 8.50196 | 0.024156 | down |
| C17orf51 | 1.17283 | 4.17695 | 0.029065 | down |
| BUB1B | 4.9479 | 17.8518 | 0.004024 | down |
| BTBD17 | 20.554 | 67.3667 | 0.004024 | down |
| BRI3BP | 1.17725 | 2.92743 | 0.004024 | down |
| BRD7 | 3.21291 | 7.17773 | 0.016692 | down |
| BORCS7 | 4.5105 | 9.92967 | 0.007147 | down |
| BIRC5 | 9.20832 | 27.4716 | 0.004024 | down |
| BCKDHB | 2.10019 | 6.45671 | 0.004024 | down |
| ATM | 2.26845 | 6.70145 | 0.0186 | down |
| ATF6B | 4.0478 | 11.4665 | 0.009746 | down |
| ATF6B | 10.2102 | 28.1058 | 0.004024 | down |
| ASPM | 2.5908 | 5.48646 | 0.004024 | down |
| ASF1B | 2.93391 | 9.67006 | 0.004024 | down |
| ARSD | 0.714145 | 1.83658 | 0.048958 | down |
| ARHGAP11A | 1.50988 | 5.25715 | 0.0186 | down |
| ARGLU1 | 27.5512 | 88.505 | 0.007147 | down |
| ARGLU1 | 30.076 | 76.7663 | 0.004024 | down |
| AP000662.4 | 1.3457 | 7.71129 | 0.040714 | down |
| ADCY6 | 3.31581 | 11.1472 | 0.004024 | down |
| ADAMTSL1 | 3.89806 | 7.80149 | 0.016692 | down |
| ACBD7 | 4.55169 | 14.3939 | 0.004024 | down |
| AC004540.4 | 16.3276 | 40.5446 | 0.004024 | down |
| AAAS | 7.52407 | 18.1407 | 0.040714 | down |

**Table S4. Expression level of DE ISOs between ZKV infected and mock infected samples**. ISO were significant differentially expressed in ZIKV infected hNPCs compared to control. “Zika” and “Control” column denote FPKM value. “up” and “down” denotes upregulation and downregulation, respectively. “isoform” column denotes gene isoforms.

**Table S5.**

| gene | Location | Adjusted_PValue | SplicingType |
| --- | --- | --- | --- |
| DCC | 18:53391655-53391715 | 0.048367 | alt3 |
| HRAS | 11:533277-533358 | 0.007556 | cassette |
| CADM1 | 11:115209574-115209657 | 0.046099 | cassette_multi |
| IGF2 | 11:2137192-2137305 | 0.018677 | altstart |
| VAV2 | 9:133787246-133787260 | 0.009571 | cassette |
| FXR1 | 3:180962940-180963028 | 0.005731 | retain_intron |
| FXR1 | 3:180975313-180975404 | 0.039276 | cassette_multi |
| SLTM | 15:58899469-58899937 | 0.018756 | cassette |
| APP | 21:26000015-26000182 | 3.75E-05 | cassette_multi |
| ATXN3 | 14:92079415-92079468 | 0.019911 | cassette |
| UNC5B | 10:71288958-71288990 | 0.011153 | cassette |
| BNIP2 | 15:59668103-59668138 | 0.009678 | cassette_multi |
| TIA1 | 2:70229059-70229091 | 0.037827 | cassette_multi |
| TIAL1 | 10:119576847-119576905 | 0.001063 | cassette |
| TIAL1 | 10:119582557-119582608 | 0.017764 | alt3 |
| MAPK8 | 10:48325897-48325965 | 0.027192 | cassette |
| RNF34 | 12:121402742-121402807 | 0.04351 | cassette |
| CASP2 | 7:143289588-143289656 | 0.036055 | altstart |
| ZIM2 | 19:56826388-56826463 | 0.00556 | cassette |

**Table S5. Alternatively spliced genes were enriched in cell death.** “Location” column denotes the range of AS events. “SplicingType” column denotes alternatively spliced type.

**Table S6.**

| gene | Location | Adjusted_PValue | SplicingType |
| --- | --- | --- | --- |
| AGRN | 1:1053461-1053493 | 1.30E-06 | cassette_multi |
| AGRN | 1:1051993-1052016 | 1.52E-05 | cassette_multi |
| APP | 21:26000015-26000182 | 3.75E-05 | cassette_multi |
| MAP2 | 2:209704450-209704620 | 0.001034 | cassette_multi |
| CTNNA2 | 2:80619085-80619228 | 0.00808 | cassette |
| MYO6 | 6:75911672-75911698 | 0.009276 | cassette |
| ABI2 | 2:203411285-203411371 | 0.009993 | cassette |
| UNC5B | 10:71288958-71288990 | 0.011153 | cassette |
| MACF1 | 1:39427961-39428287 | 0.01852 | cassette |
| MACF1 | 1:39436482-39436490 | 0.029678 | cassette |
| PBX3 | 9:125962102-125962214 | 0.03165 | cassette |
| PARD3 | 10:34372498-34372533 | 0.034012 | altstart |
| MAP2 | 2:209692625-209692792 | 4.89E-05 | altend |
| MAP2 | 2:209696542-209696748 | 0.002173 | altend |
| DCC | 18:53391655-53391715 | 0.048367 | alt3 |

**Table S6. Alternatively spliced genes were enriched in neuron development.** “Location” column denotes the range of AS events. “SplicingType” column denotes alternatively spliced type.

**Table S7**

| gene | Location | Adjusted_PValue | SplicingType |
| --- | --- | --- | --- |
| HNRNPD | 4:82354154-82355304 | 0.000769 | retain_intron |
| SRRT | 7:100888143-100888257 | 0.00183 | retain_intron |
| CPSF3L | 1:1312368-1312440 | 0.009962 | retain_intron |
| HNRNPC | 14:21263667-21263829 | 4.75E-06 | mutually_exclusive |
| TRA2A | 7:23522121-23522432 | 0.028558 | mutually_exclusive |
| APP | 21:26000015-26000182 | 3.75E-05 | cassette_multi |
| HNRNPD | 4:82354895-82355001 | 1.70E-10 | cassette |
| RBM6 | 3:49999440-49999513 | 0.000686 | cassette |
| RBM6 | 3:50060801-50060835 | 0.000913 | cassette |
| PCBP2 | 12:53467805-53467843 | 0.01756 | cassette |
| RBM23 | 14:22908333-22908399 | 0.019355 | cassette |
| HNRNPA2B1 | 7:26197831-26197866 | 0.020898 | cassette |
| RBM3 | x:48575815-48576083 | 0.022532 | cassette |
| TRA2A | 7:23531181-23531270 | 0.026597 | cassette |
| HNRNPD | 4:82356537-82356683 | 0.031192 | cassette |
| FIP1L1 | 4:53379073-53379117 | 0.047707 | cassette |
| ZRANB2 | 1:71065719-71065752 | 0.016968 | altend |
| KHSRP | 19:6415534-6415609 | 0.009543 | alt5 |
| RPP14 | 3:58306417-58306651 | 0.029807 | alt5 |
| RBM39 | 20:35709256-35709274 | 0.009279 | alt3 |

**Table S7. Alternatively spliced genes were enriched in RNA processing.** “Location” column denotes the range of AS events. “SplicingType” column denotes alternatively spliced type.

**Table S8.**

| gene | Location | Adjusted_PValue | SplicingType |
| --- | --- | --- | --- |
| XPO1 | 2:61493053-61493894 | 0.014471 | retain_intron |
| RPAIN | 17:5426236-5426299 | 0.018788 | mutually_exclusive |
| APP | 21:26000015-26000182 | 3.75E-05 | cassette_multi |
| RPGR | x:38287861-38288041 | 0.04691 | cassette_multi |
| ATL2 | 2:38296686-38296700 | 0.006996 | cassette |
| SEC31A | 4:82862534-82862572 | 0.008372 | cassette |
| MYO6 | 6:75911672-75911698 | 0.009276 | cassette |
| FLNA | x:154357251-154357274 | 0.0116 | cassette |
| CLTA | 9:36209267-36209320 | 0.012834 | cassette |
| ENAH | 1:225504991-225505053 | 0.01451 | cassette |
| MACF1 | 1:39427961-39428287 | 0.01852 | cassette |
| SRP9 | 1:225786862-225786985 | 0.027648 | cassette |
| MACF1 | 1:39436482-39436490 | 0.029678 | cassette |
| KLHL2 | 4:165321312-165321375 | 0.045358 | cassette |
| ERC1 | 12:1204501-1204512 | 0.049431 | cassette |
| TPM1 | 15:63048576-63048707 | 0.003503 | altstart |
| TPM1 | 15:63063804-63064142 | 0.005386 | altstart |
| KLC1 | 14:103679518-103679545 | 0.003664 | alt5 |
| SMG7 | 1:183542364-183542502 | 0.018452 | alt5 |

**Table S8. Alternatively spliced genes were enriched in transport.**

“Location” column denotes the range of AS events. “SplicingType” column denotes alternatively spliced type.

**Figure S1.**

**
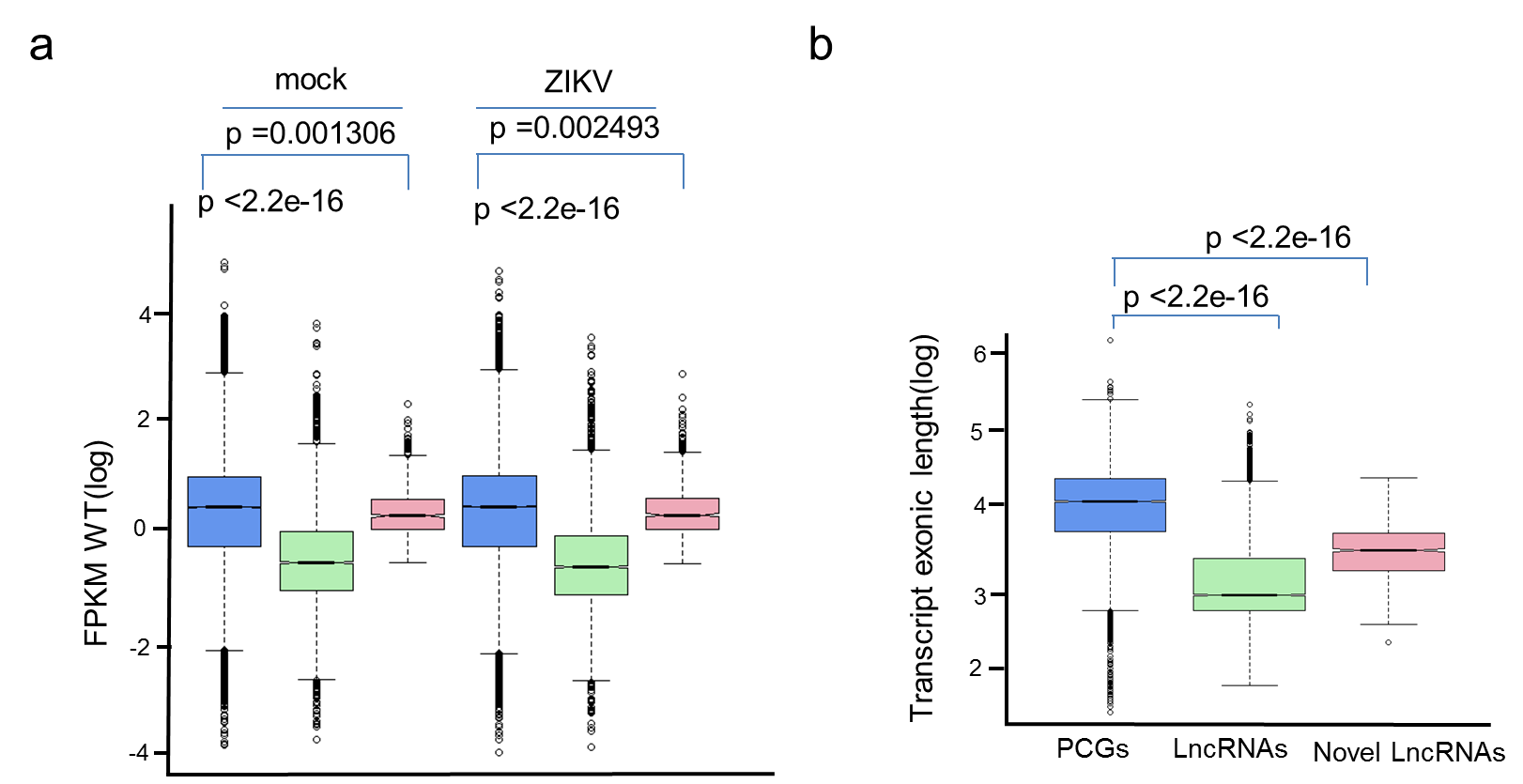
**

**Figure S1. Comparison of Novel lncRNAs with PCGs**

a. The PCGs represent higher expression level than novel lncRNAs on average; b. The novel lncRNAs show shorter length on average than PCGs .Wilcox.test, *p value* <0.05.

**Figure S2**


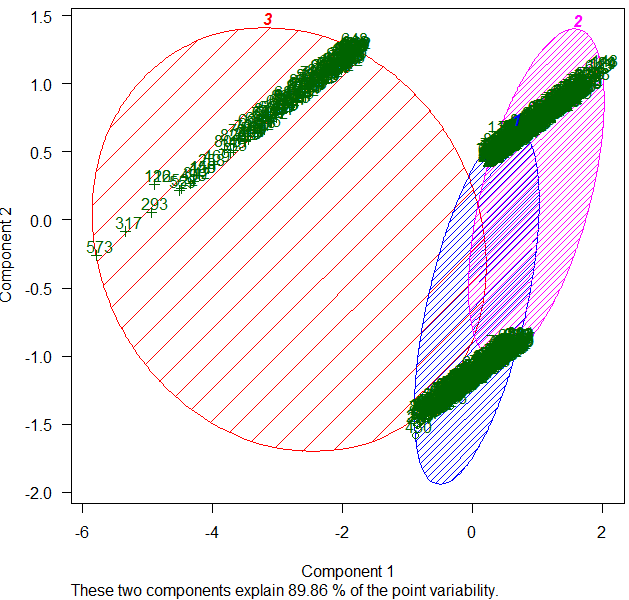


**Figure S2. The cluster of DE lncRNAs and PCGs. K-means algorithm produced 3 clusters of DE lncRNAs and PCGs. Cluster 1, 2 and 3 contained 106, 361 and 429 genes, respectively.**

**Figure S3.**

**
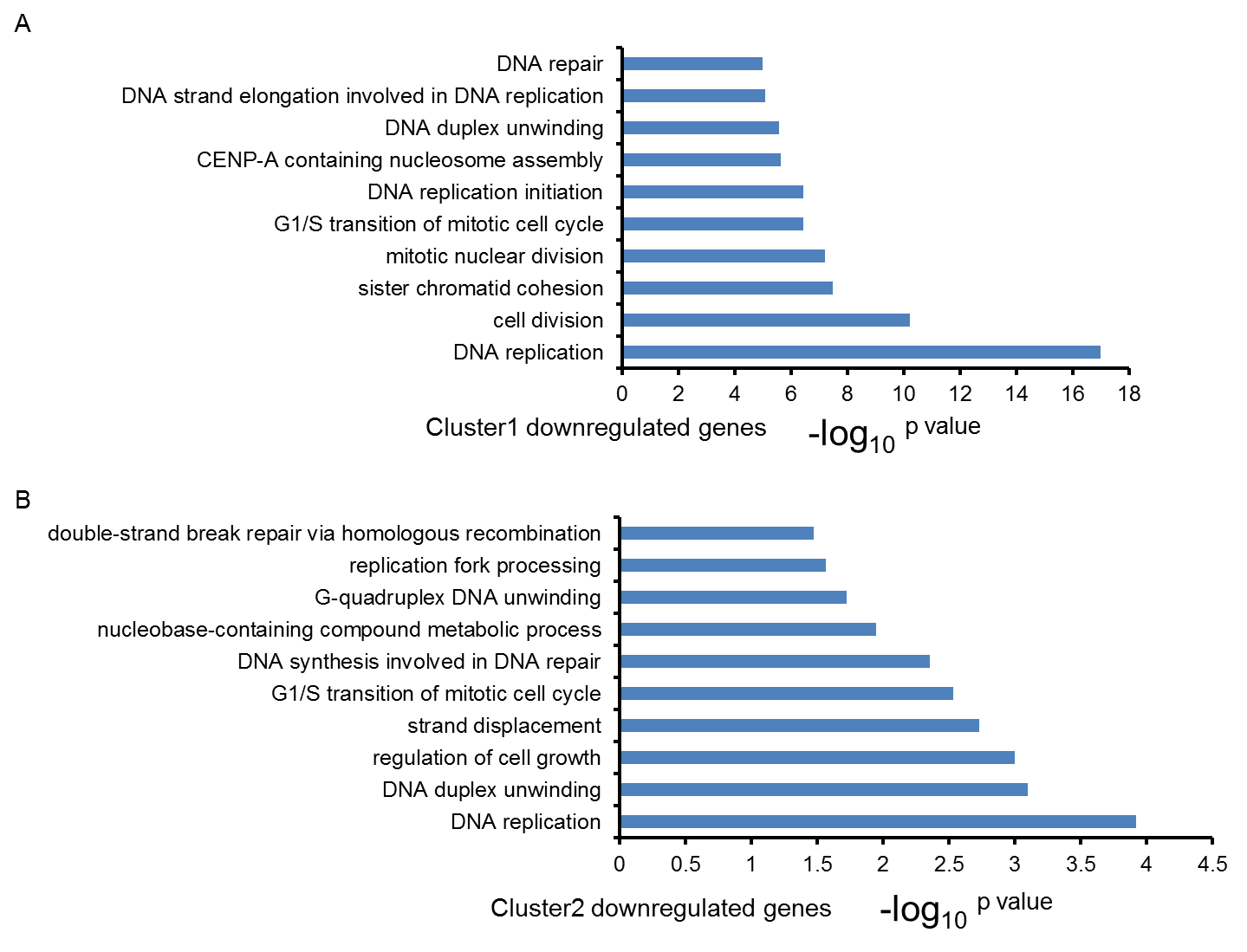

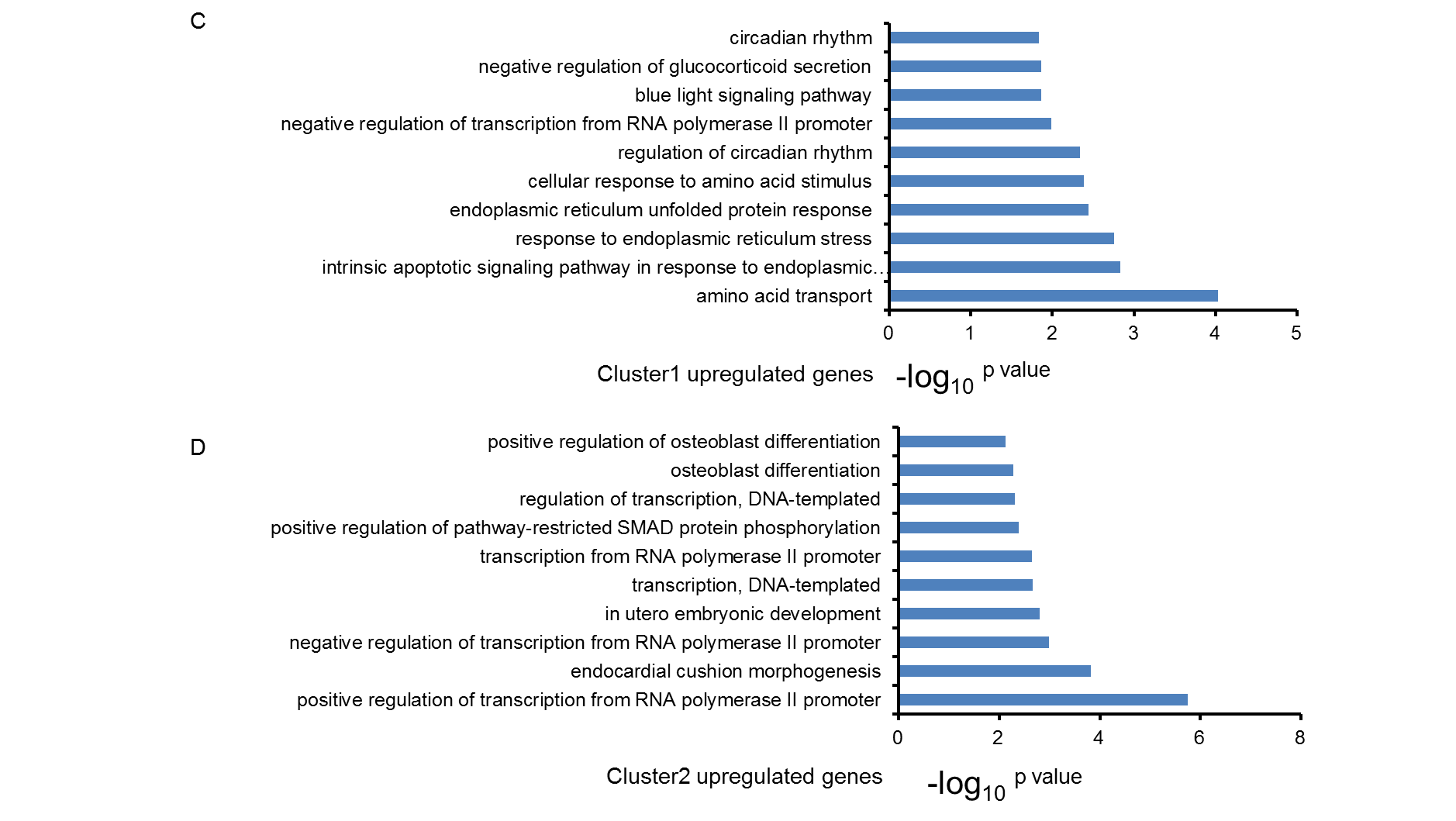
**

**Figure S3**. **GO analysis of DE genes from Cluster 1 and 2**.

(A-B): Downregulated genes from Cluster 1 and 2 were mainly enriched in DNA replication and cell cycle. Upregulated genes from Cluster 1 were enriched in amino acid transport, while genes from cluster 2 in regulation of transcription.

**Figure S4.**


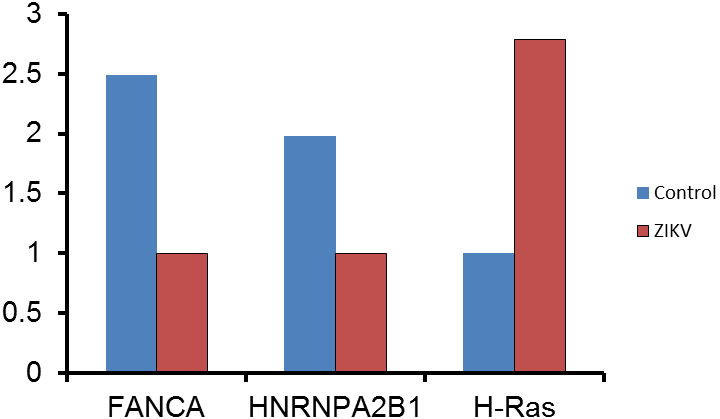


Fold change (FPKM)

🞷🞷

🞷🞷

🞷

**Figure S4**. **ZIKV infection affected the expression level of FANCA, HNRNPA2B1, H-Ras**.

Compared to control, FANCA and HNRNPA2B1 were downregulated about 2-fold by ZIKV infection, while H-Ras was upregulated about 2.5-fold in infected samples. Each group consists of two biological replicates. 🞷: *p value* <0.05, 🞷🞷: *p value* <0.01.

**Figure S5.**


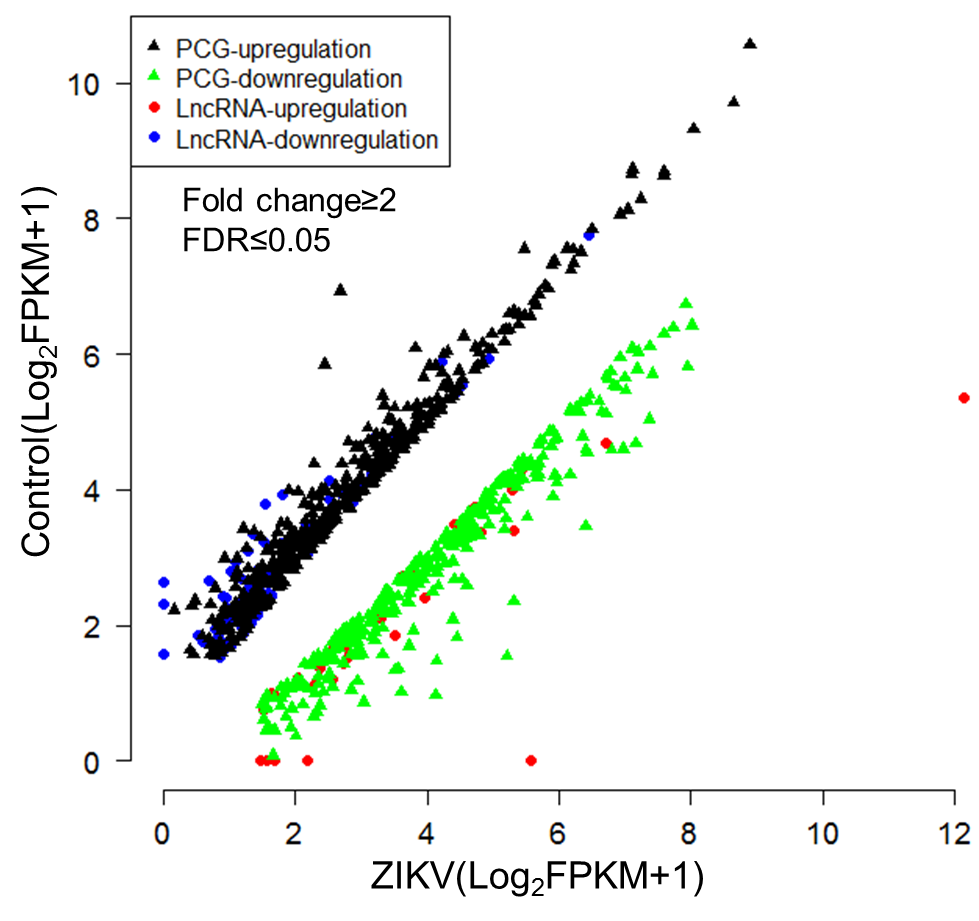


**Figure S5**. **ZIKV infection affected the expression level of all genes**.

Differentially expressed genes, including PCGs and LncRNAs, were determined by at least two-fold changes in RNA levels after Zika infection (Fold change≥2 and FDR≤0.05). A total of 896 genes were differentially expressed after infection including 540 that were upregulated and 356 downregulated.

**Figure S6**.


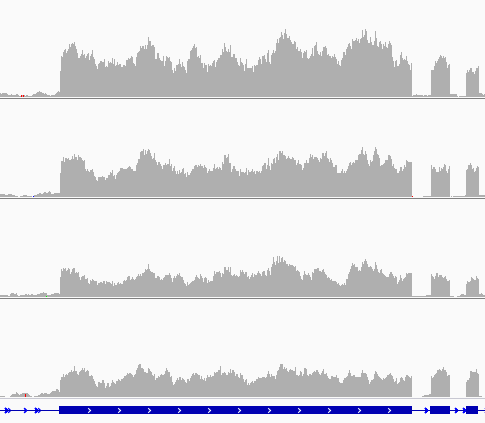


188

0

189

0

195

0

203

0

mock1

mock2

ZIKV1

ZIKV2

MAP2: altend


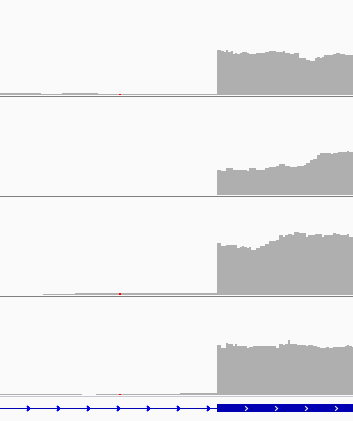


85

0

85

0

85

0

85

0

mock1

mock2

ZIKV1

ZIKV2

DCC: alt3

**Figure S6**. **ZIKV infection induced alternative splicing of neuronal genes**.

Compared to control, MAP2 and DCC were subjected to altend and alt3 by ZIKV infection, respectively.
